# Genus-specific remodeling of carbon and energy metabolism facilitates acetoclastic methanogenesis in *Methanosarcina* spp. and *Methanothrix* spp.

**DOI:** 10.1128/jb.00448-25

**Published:** 2026-01-22

**Authors:** Blake E. Downing, Dinesh Gupta, Katie E. Shalvarjian, Dipti D. Nayak

**Affiliations:** 1 Department of Plant and Microbial Biology, University of California Berkeley 1438, Berkeley, California, USA; 2 Department of Molecular and Cell Biology, University of California Berkeley 1438, Berkeley, California, USA; University of Florida, Gainesville, Florida, USA

**Keywords:** acetoclastic methanogenesis, *Methanothrix*, *Methanosarcina*, methanogen

## Abstract

Methanogenic archaea (methanogens) are microorganisms that obligately produce methane as a byproduct of their energy metabolism. While most methanogens grow on CO_2_+H_2_, isolates of the genera *Methanosarcina* and *Methanothrix* can use acetate as the sole substrate for methanogenesis. Methanogenic growth on acetate, i.e., acetoclastic methanogenesis, is hypothesized to require two distinct genetic modules: one for the activation of acetate to acetyl-CoA and another for producing a chemiosmotic gradient using electrons derived from ferredoxin. In *Methanosarcina* spp., the activation of acetate to acetyl-CoA is mediated by acetate kinase (Ack) and phosphotransacetylase (Pta), whereas *Methanothrix* spp. encode AMP-forming acetyl-CoA synthetases (Acs). The *Rhodobacter* nitrogen fixation complex (Rnf) or energy-converting hydrogenase (Ech) is critical for energy conservation in *Methanosarcina* spp. during growth on acetate, and a F_420_:methanophenazine oxidoreductase-like complex (Fpo′) likely plays an analogous role in *Methanothrix* spp. Here, we tested the proposed modularity of these pathways to facilitate acetoclastic methanogenesis. First, we surveyed over 100 genomes within the class *Methanosarcinia* to show that the genomic potential for acetoclastic methanogenesis is widespread. We then used the genetically tractable strain, *Methanosarcina acetivorans,* to build all modular combinations that might support acetoclastic methanogenesis. Our results indicate that Acs, while functional, cannot replace Ack+Pta to rescue acetate growth in *M. acetivorans*. Similarly, the Fpo′ bioenergetic complex cannot replace Rnf. As such, our work suggests that, in addition to horizontal gene transfer of core catabolic modules, acetoclastic metabolism in methanogens requires changes to core energy metabolism too.

**IMPORTANCE:**

A large fraction of biogenic methane is derived from acetate, yet acetoclastic methanogens, i.e., methanogens that grow on acetate, remain poorly characterized due to their slow growth. Two groups of methanogens, *Methanosarcina* spp. and *Methanothrix* spp., perform acetoclastic methanogenesis using distinct sets of genes for acetate activation and energy conservation. It is widely hypothesized that these genetic modules from *Methanosarcina* spp. and *Methanothrix* spp. are functionally analogous and would thus be interchangeable. To test this hypothesis, we engineered different combinations of modules for acetoclastic growth in *Methanosarcina acetivorans*. Our results challenge this hypothesized paradigm of modularity, and we posit that other changes to the carbon and electron transfer pathways are crucial for the emergence of acetoclastic methanogenesis.

## INTRODUCTION

Methane (CH_4_) is a potent greenhouse gas that traps heat in Earth’s atmosphere up to one hundred times more effectively than an equivalent amount of carbon dioxide (CO_2_) over a period of 20 years (1, 2). An accurate accounting of the sources and sinks of methane is important for modeling Earth’s climate in the past, present, and future. A large fraction of biogenic methane released into the atmosphere is produced by methanogens, microorganisms that generate methane as a by-product of their energy metabolism (1, 3). Methanogens are ubiquitous in anoxic environments ranging from sediments to the human gastrointestinal tract (3), and their growth substrates range from inorganic (H_2_+CO_2_) to organic (C_1_ compounds or acetate) depending on the environment (3–5). Currently, acetate fuels methanogenesis in human-built environments like waste-water treatment facilities and landfills, but this process evolved at least 250-million years ago when methanogens belonging to the genus *Methanosarcina* acquired genes for acetate catabolism by horizontal gene transfer from bacteria (5, 6).

**Fig 1:**
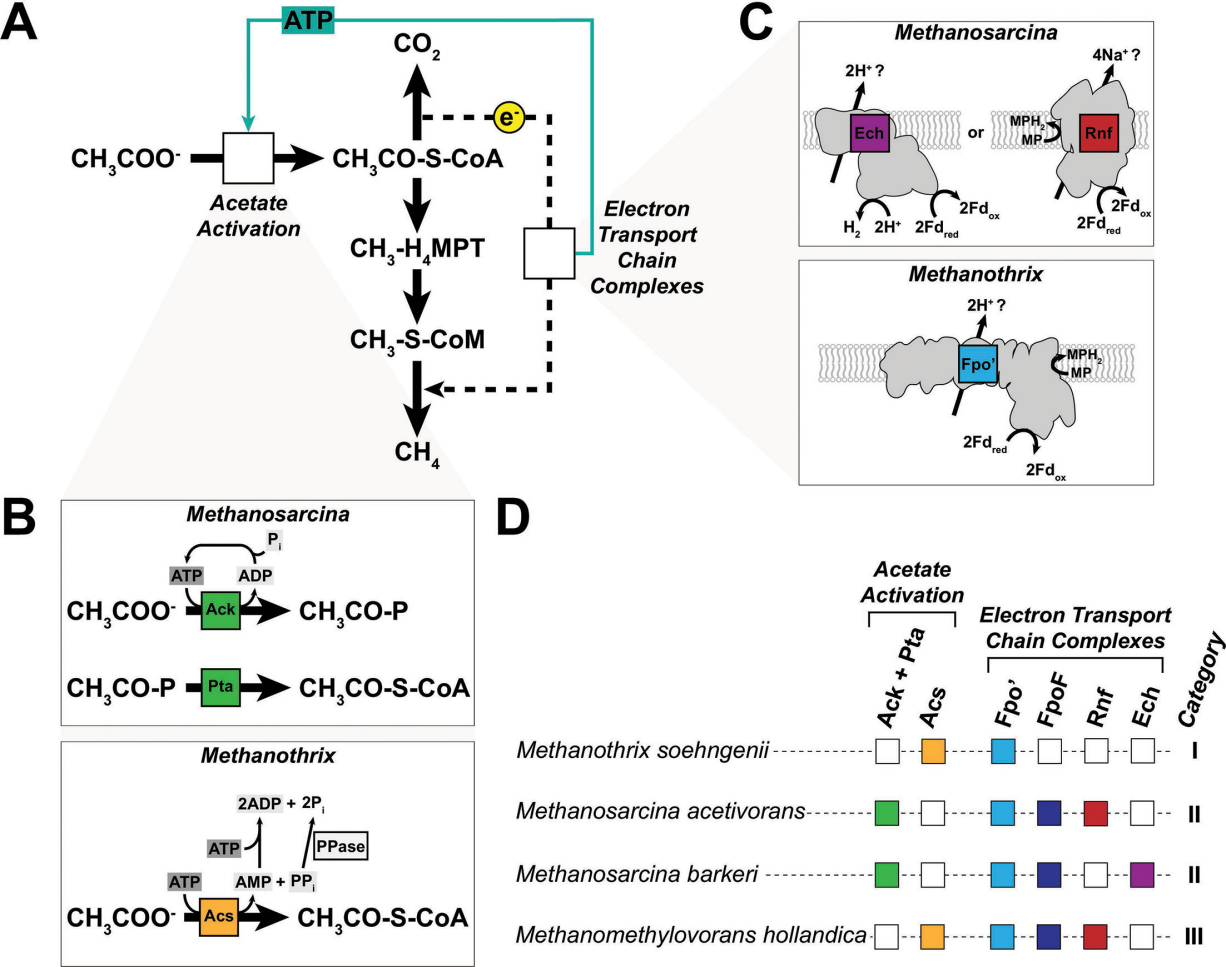
Pathways for acetate consumption and energy conservation across the class *Methanosarcinia*. (**A**) Pathways of carbon (solid arrows) and electron (dashed arrow) flow during acetoclastic methanogenesis. Acetate is first activated in an ATP-dependent manner to acetyl-CoA, which is dismutated into its methyl and carbonyl groups. The methyl group enters the methanogenesis pathway via the C1-carrier tetrahydromethanopterin (H_4_MPT) (or a derivative in select methanogens, tetrahydrosarcinopterin [H_4_SPT]) and is subsequently transferred to another C1-carrier, coenzyme M (CoM). The carbonyl group is oxidized to carbon dioxide and reducing equivalents derived from this process (e^−^) pass through the electron transport chain. The electron transport chain provides the electrons necessary for the reduction of the methyl group to methane, while simultaneously generating the ATP necessary for substrate activation (solid teal arrow). For simplicity, the carbon and electron flow steps do not show cofactors and/or electron carriers. Empty boxes labeled “Acetate Activation” and “Electron Transport Chain Complexes” note where proteins catalyzing these functions differ between methanogens. (**B**) Schematics of acetate activation in different acetoclastic methanogens. In *Methanosarcina* spp., acetate is activated in an ATP-dependent manner to acetyl-phosphate (acetyl-P) by Ack, which is then converted to acetyl-CoA through the activity of a second enzyme, Pta. The single ATP spent during activation is regenerated likely through the activity of ATP synthase or other ADP-dependent enzymes. In *Methnothrix* spp., acetate is activated directly to acetyl-CoA in a single, ATP-dependent step catalyzed by Acs coupled to the production of AMP and PP_i_. AMP is then regenerated to ADP through the hydrolysis of a second equivalent of ATP. A soluble pyrophosphatase (PPase) hydrolyzes PP_i_ to prevent accumulation of this byproduct and pull the reaction forward. (**C**) Schematics of the electron transport chain complexes involved in energy conservation during acetoclastic methanogenesis. In *Methanosarcina* spp., reduced ferredoxin generated by acetoclastic methanogenesis (Fd_red_) is re-oxidized via either the proton-translocating Ech complex coupled to hydrogen gas (H_2_) production, or the sodium-translocating Rnf complex coupled to methanophenazine (MP) reduction (MPH_2_). In *Methanothrix*, re-oxidation of Fd_red_ coupled to MP reduction is thought to be catalyzed by a proton-translocating F_420_:methanophenazine oxidoreductase homolog that lacks the coenzyme-F420 active site-containing subunit (Fpo′). (**D**) Presence/absence of acetate activation and electron transport modules in four representative methanogen species across the *Methanosarcinia*. Categories “Acetate Activation” and “Electron Transport Complexes” refer to the proteins shown in panels **B and C**, respectively. For each species, the presence or absence of the protein(s) is indicated by a color-filled or empty box, respectively: Ack+Pta (acetate kinase + phosphotransacetylase), green; Acs (acetyl-CoA synthetase [AMP-forming]), yellow; Fpo′ (F_420_:methanophenazine oxidoreductase lacking FpoF subunit), light blue; FpoF (coenzyme F_420_ active site-containing subunit of F_420_:methanophenazine oxidoreductase), dark blue; Rnf (*Rhodobacter* nitrogen fixation complex), red; Ech (energy-converting hydrogenase), purple.

Acetoclastic methanogenesis has been demonstrated in two distinct genera within the class *Methanosarcinia: Methanosarcina* and *Methanothrix* (5, 7). *Methanosarcina* spp. are metabolic generalists with a broad substrate range that includes H_2_+CO_2_, C_1_ compounds like methanol or methylamines, as well as acetate (5, 7). In contrast, *Methanothrix* spp. are metabolic specialists that can only grow on acetate (5, 7). A growth rate versus yield tradeoff likely allows these two groups of methanogens to occupy distinct ecological niches (5). *Methanosarcina* spp. have faster growth rates and are typically found in acetate-rich environments, whereas *Methanothrix* spp. have a higher substrate affinity for acetate and thrive in acetate-limited regimes (5). These ecological distinctions are thought to stem from different pathways for activating acetate to acetyl-CoA, as well as distinct modules for energy conservation in the two groups of methanogens (Fig. 1A) (5). In the genus *Methanosarcina*, acetate activation proceeds through the combined activities of two enzymes, acetate kinase (Ack) and phosphotransacetylase (Pta), where Ack hydrolyzes an ATP to activate acetate to acetyl-phosphate and Pta catalyzes the transfer of the acetyl group to coenzyme A (CoA) to produce acetyl-CoA (Fig. 1B) (5). In the genus *Methanothrix*, acetate activation to acetyl-CoA occurs in one step via the AMP-forming acetyl-CoA synthetase (Acs), which also releases inorganic pyrophosphate (PP_i_) as a by-product (5). Although PP_i_ is an energy-rich intermediate, it is likely hydrolyzed by cytosolic pyrophosphatases and not used to conserve energy (8). The regeneration of ATP from AMP requires the hydrolysis of a second molecule of ATP as shown in (Fig. 1B) (5). Hence, the Acs-dependent acetate activation pathway in *Methanothrix* spp. requires twice as much energy investment than the Ack+Pta activation pathway in *Methanosarcina* spp. The enzymes involved in the dismutation of acetyl-CoA to produce CO_2_ and methane are largely the same between the two groups of methanogens and have been reviewed elsewhere (Fig. 1A) (4, 5, 7). During acetoclastic methanogenesis electrons carried in the form of reduced ferredoxin (Fd_red_) are used to generate a chemiosmotic gradient for ATP synthesis via an electron transport chain (ETC) (3, 9). *Methanosarcina* spp. use either the *Rhodobacter* nitrogen fixation (Rnf) complex or the membrane-bound energy-converting hydrogenase (Ech) to generate a Na^+^ or a H^+^ gradient with electrons derived from Fd_red_, respectively (Fig. 1C) (9–14). Owing to their slow growth and genetic intractability, the ETC of *Methanothrix* spp. is not well-resolved (15). However, biochemical assays with crude membrane preparations indicate that a modified form of the F_420_:methanophenazine oxidoreductase (Fpo) lacking a F_420_-interacting “head” subunit, FpoF, hereafter referred to as Fpo′ uses the electrons derived from Fd_red_ to generate a proton gradient (Fig. 1C) (16).

Despite their overwhelming contribution to the global methane budget, physiological studies with acetoclastic methanogens, especially *Methanothrix* spp., have been sparse, especially in the last decade (17). Here, we assess the distribution of modules associated with acetoclastic methanogenesis in sequenced methanogen genomes and also experimentally test if the modules from *Methanosarcina* spp. and *Methanothrix* spp. are cross-compatible. First, we identify strains that might have the potential to perform acetoclastic methanogenesis using a profile hidden Markov model (HMM)-based genomic survey. While the specific combination of acetate activation genes and energy conservation modules found in *Methanothrix* spp. or *Methanosarcina* spp. is absent in other genomes, alternate combinations, especially Acs and Rnf, are more broadly distributed. We then engineered strains of *Methanosarcina acetivorans* with different combinations of activation pathways (Ack+Pta versus Acs) and bioenergetic modules (Rnf/Ech versus Fpo′) that might fuel acetoclastic methanogenesis. Our findings suggest that existing combinations of acetate activation and energy conservation are intricately linked, such that these two modules must co-evolve to facilitate methanogenic growth on acetate.

## RESULTS

### Genomic analysis highlights the potential for novel acetoclastic methanogenesis pathways

We used a custom, profile HMM-based search tool to survey extant methanogens for the presence of proteins that play a role in either acetate activation or energy conservation during acetoclastic methanogenesis. We restricted our search to sequenced genomes available through the Genome Taxonomy Database (GTDB r214.0) within the class *Methanosarcinia* (*n* = 133 genomes) as it comprises most known methanogens with an ETC, which is essential for energy conservation during acetoclastic growth.

First, we surveyed proteins involved in acetate activation, either Ack+Pta or Acs. We found that Ack+Pta is present in *Methanosarcina* spp. (94%, *n* = 29/31, Fig. S1, Table S1) and in some members of the genus *Methanimicrococcus* (50%, *n* = 3/6), including the type strain *Methanimicrococcus blatticola* (18). In contrast, Acs is more broadly distributed as we found hits within *Methanothrix* (89%, *n* = 24/27), *Methermicoccus* (100%, *n* = 1), *Methanomethylovorans* (86%, *n* = 6/7), and multiple other genera (Fig. S1, Table S1). We did not detect the co-occurrence of Ack+Pta and Acs in any of the sequenced genomes.

Next, we searched for energy conservation modules that can use Fd_red_ to generate a chemiosmotic gradient, i.e., Rnf, Ech, or Fpo′ (9, 13). Rnf is broadly distributed in several different genera, including *Methanosarcina* (26%, *n* = 8/31), *Methanolobus* (100%, *n* = 19/19), and *Methanomethylovorans* (86%, *n* = 6/7) (Fig. S1, Table S1). However, we were only able to detect Ech in members of *Methanosarcina* (71%, *n* = 22/31, Fig. S1, Table S1). Since the canonical Fpo complex interacts with F_420_ via FpoF (19), we surveyed genomes for all subunits of the Fpo complex excluding FpoF (Fpo′), and separately searched for FpoF to distinguish between these potential variants. While Fpo (Fpo′+FpoF) is broadly distributed within the class *Methanosarcinia* (56%, *n* = 75/133, Fig. S1, Table S1), genomes that solely encode Fpo′ are more limited (26%, *n* = 34/133) and are typically restricted to members of the genus *Methanothrix* (81%, *n* = 22/27) and *Methermicoccus* (100%, *n* = 1, Fig. S1, Table S1).

Based on our survey, we developed a classification scheme to describe patterns of co-occurrence between acetate activation and energy conservation modules (Fig. 1D). We define the category I genomes as those containing Acs for acetate activation and Fpo′ for energy conservation (23% of *Methanosarcinia*, *n* = 30/133), as exemplified by *Methanothrix soehngenii*. Category II genomes include those that use Ack+Pta to activate acetate, and either Rnf or Ech to conserve energy (25% of *Methanosarcinia*, *n* = 33/134). Most category II genomes also encode a complete Fpo complex (82% of category II, *n* = 27/33). Category II is represented by genomes such as *M. acetivorans* and *Methanosarcina barkeri* (Fig. 1D). We define category III genomes as those containing the category I acetate activation module (Acs) alongside the category II energy conservation modules (i.e., Rnf), thus representing a hybrid between the first two categories (32% of *Methanosarcinia*, *n* = 42/133) (Fig. 1D). *Methanomethylovorans hollandica* represents a category III genome. Notably, acetoclastic methanogenesis has not yet been demonstrated in a category III strain. Intriguingly, we did not find a single genome that encodes Ack+Pta and just Fpo′, another potential combination of modules that might facilitate acetoclastic methanogenesis. Our analysis raises questions about the cross-compatibility between substrate activation and energy conservation modules in acetoclastic methanogens. In other words, even though the acetate activation and energy conservation modules perform the same biochemistry, are they functionally analogous and interchangeable?

### Acetyl-CoA synthetases are functional but do not support acetoclastic growth of *M. acetivorans*

**Fig 2:**
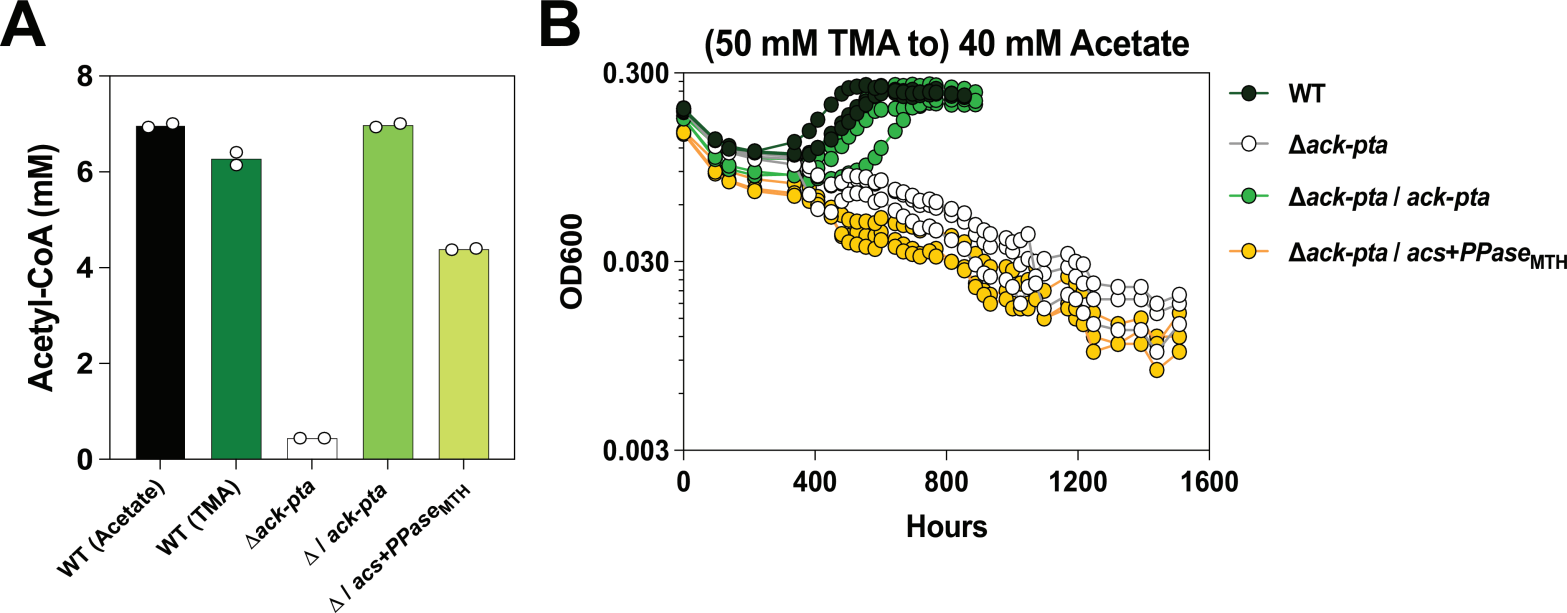
Complementation of the *M. acetivorans ack-pta* deletion mutant with *acs* does not restore growth on acetate. (**A**) Quantification of acetyl-CoA production from cell lysates using the acetylhydroxamate assay. Assays were conducted with WWM60 (wild type or WT) grown in high-salt (HS) minimal medium with 40 mM acetate (black bar) or 50 mM trimethylamine (TMA) (dark green bar), the ∆*ack-pta* mutant (white bar), the ∆*ack-pta* expressing *ack-pta* in *trans* (∆*ack-pta/*P*_mcrB_*_(tetO1)_*-ack-pta*) (light green bar), and the ∆*ack-pta* mutant expressing *acs+PPase*_MTH_ in *trans* (∆*ack-pta/*P*_mcrB_*_(tetO1)_*-acs+PPase*_MTH_) (yellow-green bar) were grown in HS medium with 50 mM TMA and 2 µg/mL Puromycin was added to maintain the complementation plasmid (if present). All reactions were performed with two biological replicates. Each replicate comprised 1 mg total protein that was incubated at 37°C for 30 minutes under the assay conditions (see Materials and Methods). (**B**) Growth curves of WT (dark green circles), the *∆ack-pta* mutant (white circles), and complemented strains ∆*ack-pta*/*ack-pta* (light green circles) and *∆ack-pta/acs+Ppase*_MTH_ (yellow circles). Cells were pre-grown in HS with 50 mM TMA and inoculated into HS medium with 40 mM acetate. 2 µg/mL Puromycin was added to the growth medium for maintenance of the complementation plasmid (if present) and 100 µg/mL tetracycline for full induction of the complemented genes. Three replicate growth curves were conducted for each strain. Growth parameters for each strain are shown in Table 1.

First, we explored the possibility of acetoclastic methanogenesis in category III strains (Fig. 1D). Theoretically, these methanogens could grow on acetate using Acs for acetate activation and conserve energy using the Rnf complex. To test this possibility, we chose to engineer *M. acetivorans,* a category II strain, into a category III strain rather than test for acetate growth in a naturally occurring category III methanogen. We opted for the former because acetate growth under laboratory conditions is well established in *M. acetivorans*. As the first step in the engineering process, we deleted the native acetate activation module (∆*ack-pta*, MA3606-3607, MA_RS18805-18810) in the parent strain (WWM60, referred to as wild type or WT). We verified the markerless chromosomal deletion of the *ack-pta* genes, as well as the absence of any off-target mutations due to CRISPR editing by whole-genome sequencing (Table S2). To validate the absence of Ack and Pta, we also assayed for acetyl-CoA production in cell lysates using the acetylhydroxamate assay as previously described (20, 21). The ∆*ack-pta* mutant had *ca*. 7% of the activity observed in the WT strain, which did not seem to vary in a substrate-dependent manner (Fig. 2A). The ∆*ack-pta* mutant was also incapable of growth on acetate as the sole substrate for methanogenesis, even after prolonged incubation for 6+ months (Fig. 2B, Table 1). Curiously, despite the ability to generate acetyl-CoA on trimethylamine (TMA), WT does not have a detectable growth advantage in minimal medium containing both TMA and acetate compared to the ∆*ack-pta* mutant (Fig. S2, Table S3). To further corroborate that the production of acetyl-CoA in WT is due to the activity of Ack+Pta, we complemented the ∆*ack-pta* mutant in *trans* with a plasmid expressing the native *ack* and *pta* locus from a tetracycline-inducible promoter (∆*ack-pta/*P*_mcrB_*_(tetO1)_*-ack-pta*). Acetyl-CoA production and acetate growth were restored in the complementation mutant (Fig. 2, Table 1).

**TABLE 1:** Growth characteristics of the WWM60 (wild type or WT), the ∆*ack-pta* mutant and the ∆*ack-pta*/*ack-pta*, *∆ack-pta/acs+Ppase*_MTH_ complementation strains in HS medium supplemented with 40 mM acetate^*a*^

Substrate	Strain	T_D_ (h)^*b*^	T_D_ *P* value	T_Lag_ (h)	T_Lag_ *P* value
40 mM acetate	WT	164.3 ± 5.1	–^*c*^	498.6 ± 51.9	–
	*∆ack-pta*	n.g.	–	n.g.	–
	*∆ack-pta/ack-pta*	141.2 ± 22.7	0.23	579.5 ± 80.9	0.2
	*∆ack-pta/acs+PPase*_MTH_	n.g.	–	n.g.	–

^*a*^ Doubling times (T_D_) and lag times (T_Lag_) are reported in hours ± standard deviation. Significant difference between the T_D_ and T_Lag_ of various mutant strains compared to the WT was determined by calculating *P* values using a two-sided t-test assuming unequal variance. If no increase in optical density at 600 nm was observed after 6 months, we assumed that there was no growth. n.g., no growth.

^*b*^ The typical doubling time for WWM60 on HS+acetate is ~80 h. However, given that our *ack-pta* complementation strain demonstrates a similar doubling time, we attribute the slower growth phenotypes in this experiment to batch-specific effects in the HS media. Furthermore, given that we do not compare or claim significance across experiments, these batch-specific effects on growth are well-controlled within this experiment.

^*c*^ – indicates that a *P*-value was not calculated.

Next, we expressed Acs homologs from *M. soehngenii*, *Methanothrix harundinacea*, and *M. hollandica* in *M. acetivorans*. All the catalytically important residues and substrate-coordination motifs are conserved in the Acs sequences we selected for this study (Fig. S3) (22). First, we expressed these genes in WT and did not observe any atypical growth upon induction (Table S4). These data suggest that the expression of Acs, in and of itself, is not toxic in *M. acetivorans*. However, when we complemented the ∆*ack-pta* strain with each of these Acs homologs, no observable growth on acetate was detected, even after 6+ months of incubation (Table S4). Since Acs hydrolyzes ATP to AMP and PP_i_, we reasoned that PP_i_ buildup might limit growth in *M. acetivorans*. So, we generated additional constructs expressing Acs in conjunction with a pyrophosphatase (PPase) from *M. harundinacea* (Table S4) (23). Furthermore, we also measured acetyl-CoA production in the strain expressing *acs* and *PPase* from *M. harundinacea* (*∆ack-pta/*P*_mcrB_*_(tetO1)_*-acs+PPase*_MTH_). While we could detect 10-fold higher acetyl-CoA production in the Acs-encoding cell lysates relative to the ∆*ack-pta* mutant (Fig. 2A), even maximum induction of Acs and PPase did not restore growth on acetate (Fig. 2B, Table 1; Table S4).

Finally, we hypothesized that the bifunctional acetyl-CoA decarbonylase synthase enzyme (ACDS), which catalyzes the dismutation of acetyl-CoA, might not be expressed as highly on TMA or in the absence of Ack+Pta on acetate. To test this hypothesis, we obtained the expression profiles of the two native *cdh* operons (encoding ACDS) in WT on acetate using RNA-sequencing and compared our data to a previously published data set for the same strain on TMA (12). The native expression levels of both *cdh* operons on acetate [average log_2_(FPKM) = 9.38 for *cdh1* and average log_2_(FPKM) = 10.28 for *cdh2*] were higher than on TMA [average log_2_(FPKM) = 7.58 and 7.74 for *cdh1* and *cdh2,* respectively]. Our data corroborated previous reports (24) and prompted us to experimentally test if insufficient *cdh* expression might be preventing growth on acetate in the Acs-encoding strains. Thus, we modified the plasmid encoding Acs and PPase from *M. harundinacea* to constitutively express the native *cdh2* operon from *M. acetivorans*, which has been shown to be necessary for optimal acetate growth (24), from the *Methanosarcina mazei* P*_mcrB_* promoter (∆*ack-pta*/P*_mcrB_*_MM_*-cdh2,* P*_mcrB_*_(tetO1)_*-acs+PPase*_MTH_). However, even with constitutively high expression of ACDS, no growth was detected on acetate (Table S4).

Since nearly every category III methanogen also encodes the Fpo complex (40/42 genomes) (Fig. S1, Table S1), we posited that this complex might have a role in energy conservation during acetoclastic methanogenesis. Since Rnf is likely the preferred enzyme catalyzing the re-oxidation of Fd_red_ in *M. acetivorans,* we expressed the Acs and PPase from *M. harundinacea* in the ∆*mmcA-rnf* mutant (*∆mmcA-rnf/*P*_mcrB_*_(tetO1)_*-acs+PPase*_MTH_). In the ∆*mmcA-rnf* mutant background, Fpo is the only known ion-translocating bioenergetic complex that can interact with Fd_red_ and is also upregulated by 1- to 3-fold (12). However, this mutant was also not viable on acetate (Table S4). Together, our results indicate that, despite being functional, Acs alone, or with a PPase, cannot replace Ack+Pta as an acetate activation module in *M. acetivorans*.

### Overexpression of Fpo′ does not restore acetoclastic growth in the ∆*mmcA-rnf* mutant

Unlike category III strains that are commonly found, a putative category IV strain, i.e., one encoding Ack+Pta and Fpo′, is yet to be discovered. This strain could, in principle, grow on acetate. *M. acetivorans* and many other strains that belong to the category II acetoclastic methanogens encode all the *fpo* genes in addition to Rnf (or Ech) (Fig. 1). Thus, if Fpo′ were to be made in *M. acetivorans,* it could compensate for Rnf during growth on acetate, but this likely does not happen due to regulatory constraints. Our hypothesis is based on transcriptomic analyses, which indicate that all the *fpo* genes in *M. acetivorans* have significantly lower expression on acetate [average log_2_(FPKM) = 4.83] compared to our previously reported values on TMA [average log_2_(FPKM) = 6.45] (12). To test our hypothesis, we generated two classes of mutants that would specifically overexpress some or all of the *fpo* genes on acetate.

**Fig 3:**
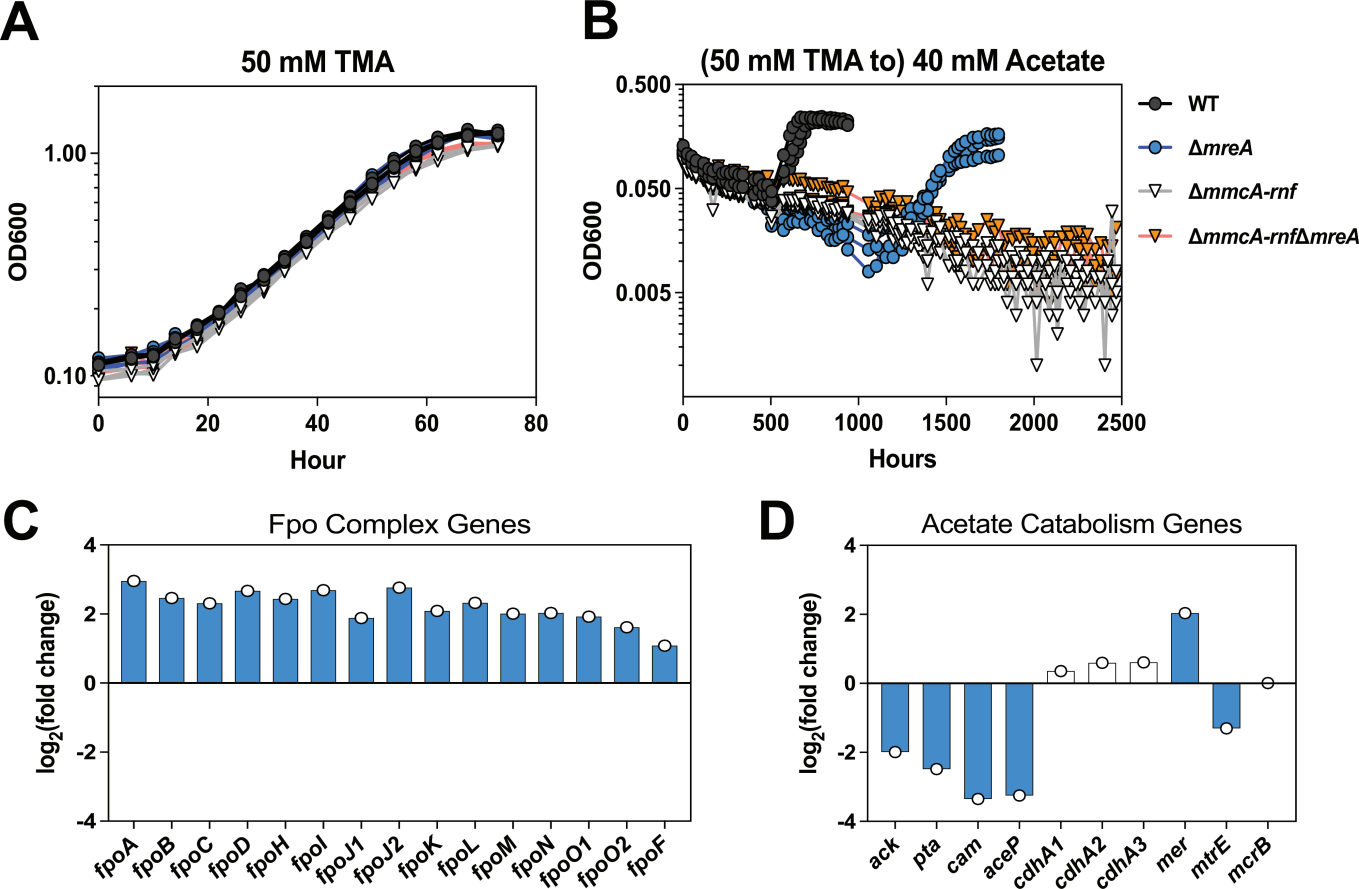
Deletion of *mreA* increases the expression of the *fpo* genes but does not permit acetate growth in the absence of the Rnf complex. Growth curve of WWM60 (wild type or WT, black circles), ∆*mreA* (blue circles), ∆*mmcA-rnf* (white inverted triangles), and ∆*mmcA-rnf*∆*mreA* (orange inverted triangles) strains in high-salt (HS) minimal medium with (**A**) 50 mM TMA or (**B**) 40 mM acetate after pre-culture in medium with 50 mM TMA. Four replicates were used for growth assays on TMA, and three replicates were used for growth assays on acetate. (**C**) Log_2_(fold change) in expression of the *fpo* genes in the ∆*mreA* mutant compared to WT during growth on acetate. Genes with higher expression in the ∆*mreA* mutant have a positive log_2_(fold change) value. (**D**) Log_2_(fold change) in expression of metabolic genes in the ∆*mreA* mutant compared to WT during growth on acetate. For catabolic enzymes composed of more than one subunit, only expression of the first gene in the operon is shown for simplicity. Genes with higher expression in the ∆*mreA* mutant have a positive log_2_(fold change) value. Genes with lower expression in the ∆*mreA* mutant have a negative log_2_(fold change) value. Statistically significant changes in gene expression (q-value ≤ 0.01) are shown in blue bars. Statistically insignificant changes in gene expression (q-value > 0.01) are shown in white bars. Gene abbreviations: *fpo*, F420:methanophenazine oxidoreductase; *ack*, acetate kinase; *pta*, phosphotransacetylase; *cam*, carbonic anhydrase; *aceP*, acetate permease; *cdh*, carbon monoxide dehydrogenase/acetyl-CoA synthase; *mer*, methylenetetrahydromethanopterin reductase; *mtr*, H_4_MPT:CoM methyltransferase; *mcr*, methyl coenzyme M reductase. Growth parameters for each strain are shown in Table 2.

First, we targeted a known repressor of the *fpo* genes, *mreA* (MA3302, MA_RS17255) (25). We deleted *mreA* in the wild-type background and in the ∆*mmcA-rnf* mutant to generate ∆*mreA* and ∆*mmcA-rnf*∆*mreA* mutants, respectively, and verified these mutants by whole-genome sequencing (Table S2 and S5). The growth pattern of the *∆mreA* and ∆*mmcA-rnf*∆*mreA* mutants phenocopied their parent strains on TMA (Fig. 3A, Table 2). The ∆*mreA* mutant could still grow on acetate, albeit with a substantial fitness defect (Fig. 3B, Table 2), whereas the ∆*mmcA-rnf*∆*mreA* strain was unable to grow even after 6+ months of incubation (Fig. 3B, Table 2). We also performed RNA sequencing of WT and the ∆*mreA* mutant on acetate to confirm that the expression of all the *fpo* genes is, indeed, elevated when MreA is absent (Fig. 3C). In line with previous observations, we also observed significantly lower expression of acetate kinase (*ack*) and phosphotransacetylase (*pta*) in the ∆*mreA* mutant (Fig. 3D), which could be the reason for the fitness defect observed during growth on acetate (Fig. 3B, Table 2) (25).

**TABLE 2:** Growth characteristics of WWM60 (wild type or WT), ∆*mreA*, ∆*mmcA-rnf*, ∆*mmcA-rnf*∆*mreA*, P*_mcrB_*_(tetO4)_*-fpo*, and ∆*mmcA-rnf* P*_mcrB_*_(tetO4)_*-fpo* strains in HS minimal media with either 50 mM TMA or 40 mM acetate^*a*^

Substrate	Strain	T_D_ (h)	T_D_ *P* value	T_Lag_ (h)	T_Lag_ *P* value
50 mM TMA	WT	14.2 ± 0.3	–^*b*^	11.6 ± 0.8	–
	∆*mreA*	13.8 ± 0.4	0.45	13.0 ± 0.4	0.03
50 mM TMA	∆*mmcA-rnf*	15.8 ± 0.4	–	7.7 ± 2.1	–
	∆*mmcA-rnf*∆*mreA*	15.4 ± 0.1	0.15	9.4 ± 0.4	0.23
40 mM acetate	WT	80.0 ± 2.4	–	619.4 ± 36.1	–
	∆*mreA*	133.0 ± 29.9	0.09	1589.5 ± 25.4	2.86E-6
	∆*mmcA-rnf*	n.g.	–	n.g.	–
	∆*mmcA-rnf*∆*mreA*	n.g.	–	n.g.	–
50 mM TMA	WT	14.0 ± 0.2	–	13.9 ± 2.1	–
	P*_mcrB_*_(tetO4)_-*fpo* 25 TET	14.5 ± 0.4	0.05	13.4 ± 2.6	0.73
	P*_mcrB_*_(tetO4)_-*fpo* 0 TET	n.d.	–	n.d.	–
40 mM acetate	WT	105.9 ± 12.6	–	439.3 ± 73.3	–
	P*_mcrB_*_(tetO4)_-*fpo* 25 TET	106.6 ± 3.7	0.93	485.7 ± 51.8	0.42
	P*_mcrB_*_(tetO4)_-*fpo* 0 TET	86.2 ± 5.5	0.09	534.6 ± 76.2	0.15
	∆*mmcA-rnf*	n.g.	–	n.g.	–
	∆*mmcA-rnf* P*_mcrB_*_(tetO4)_-*fpo* 25 TET	n.g	–	n.g.	–
	∆*mmcA-rnf* P*_mcrB_*_(tetO4)_-*fpo* 0 TET	n.g.	–	n.g.	–

^*a*^ Doubling times (T_D_) and lag times (T_Lag_) are reported in hours ± standard deviation. Significant difference between the T_D_ and T_Lag_ of various mutant strains compared to the WT was determined by calculating *P* values using a two-sided t-test assuming unequal variance. In each experiment, the reported *P* values are determined by comparing the mutant strain against its respective parent strain and are not measured across experiments. If no increase in optical density at 600 nm was observed after 6 months, we assumed that there was no growth. n.g., no growth. T_D_ and T_Lag_ were not determined for strains/conditions that did not grow exponentially. n.d., not determined.

^*b*^ – indicates that a *P*-value was not calculated.

**Fig 4:**
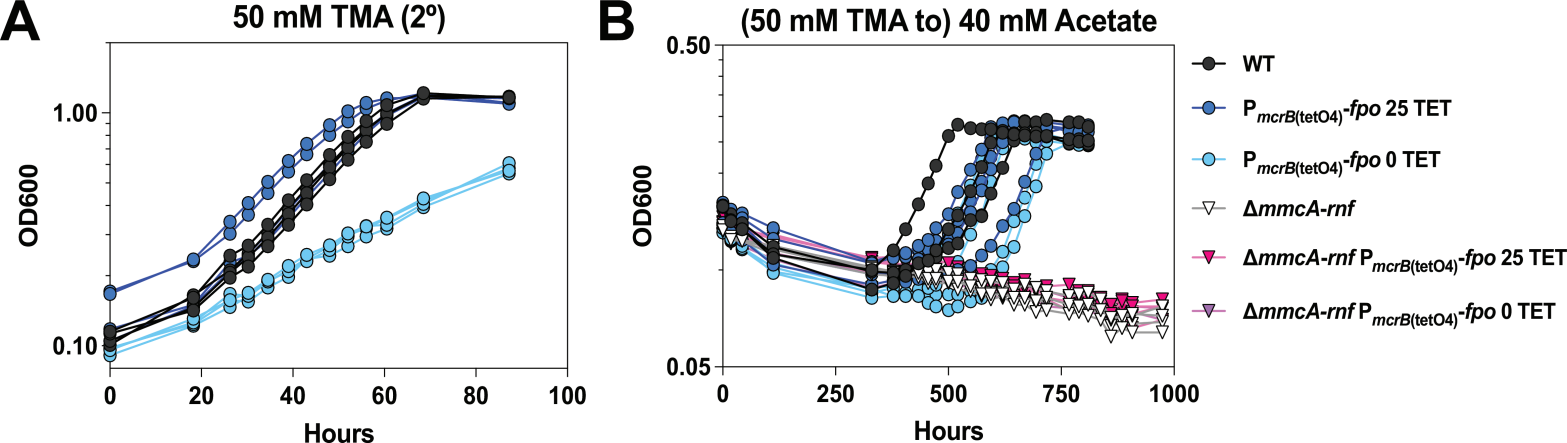
Overexpression of the *fpo*′ genes does not rescue acetoclastic growth of the ∆*mmcA-rnf* mutant. (**A**) Growth curves of WWM60 (wild type or WT, black circles) and the P*_mcrB_*_(tetO4)_*-fpo* mutant (blue circles) in high-salt (HS) minimal medium with 50 mM TMA. The P*_mcrB_*_(tetO4)_*-fpo* mutant is supplemented with 25 µg/mL tetracycline (dark blue circles) or 0 µg/mL tetracycline (light blue circles). Growth assays were conducted with cultures pre-grown in HS with 50 mM TMA with the aforementioned concentrations of tetracycline and thus are depicted as 2°. (**B**) Growth of WT (black circles), the P*_mcrB_*_(tetO4)_*-fpo* mutant strain supplemented with 25 µg/mL tetracycline (dark blue circles) or 0 µg/mL tetracycline (light blue circles), ∆*mmcA-rnf* (white triangles), and ∆*mmcA-rnf* P*_mcrB_*_(tetO4)_*-fpo* mutant strains provided with 25 µg/mL tetracycline (pink triangles) or 0 µg/mL tetracycline (purple triangles) in HS minimal medium with 40 mM acetate after pre-culture in HS medium with 50 mM TMA. Four replicate tubes for each strain were used for growth assays. Note: One replicate for WT broke during the experiment, and all data for that replicate have been omitted. Growth parameters for each strain are shown in Table 2.

Since the deletion of MreA downregulates acetate activation genes and leads to a global transcriptional response (Fig. S4; Table S6) (25), we designed an alternate strategy for targeted overexpression of the native Fpo′ in *M. acetivorans*. The Fpo complex is expected to contain 13 subunits, 12 of which are encoded as a single ~10.5 kb operon, *fpoABCDHJ_1_J_2_KLMNO_1_* (MA1495-1507, MA_RS07760-07820) (19). An additional FpoO subunit, *fpoO2* (MA1509, MA_RS25205), is encoded nearby, but its role is undefined (19). One additional subunit, FpoF (MA3732, MA_RS19445), the F_420_ interacting “head” of the complex, is found elsewhere in the genome (19). In obligately acetoclastic methanogens, the *fpoF* locus is absent, which likely enables the rest of the Fpo complex to operate as a ferredoxin:methanophenazine oxidoreductase (15). Accordingly, we hypothesized that increasing expression of the native *fpoA-O_1_* operon, without changing the expression of the *fpoF* gene, might generate more of the Fpo′ complex that could interface with Fd_red_ produced during acetoclastic growth. To this end, we replaced the native promoter of the *fpoA-O*_1_ operon with a tetracycline-inducible promoter [P*_mcrB_*_(tetO4)_] in both WT [P*_mcrB_*_(tetO4)_-*fpo*] and the ∆*mmcA-rnf* mutant [∆*mmcA-rnf* P*_mcrB_*_(tetO4)_-*fpo*] (Fig. S5A and B; Table S2 and S5). To validate tetracycline-inducible control of the native *fpoA-O*_1_ operon, we grew the P*_mcrB_*_(tetO4)_-*fpo* strain in TMA with varying concentrations of tetracycline and observed linear growth at 0 µg/mL tetracycline, indicating that the mutant becomes limited for Fpo (26, 27), which is known to be essential for growth on TMA (Fig. 4A, Table 2) (9, 13). In the ∆*mmcA-rnf* P*_mcrB_*_(tetO4)_-*fpo* mutant, which lacks the TetR repressor, the promoter swap results in constitutive expression of the native *fpoA-O_1_* operon and therefore does not limit growth on TMA (Fig. S6; Table S7). When we transferred these mutants to acetate, the P*_mcrB_*_(tetO4)_-*fpo* strain could still grow, but the ∆*mmcA-rnf* P*_mcrB_*_(tetO4)_-*fpo* mutant was incapable of growth after 6+ months of incubation (Fig. 4B, Table 2). Overall, our results indicate that increasing the expression of the native Fpo′ complex in *M. acetivorans* cannot compensate for the Rnf complex during growth on acetate.

## DISCUSSION

Our bioinformatic screen of methanogens within the class *Methanosarcinia* suggested that the genomic potential for acetoclastic methanogenesis is broader in scope than the two genera where this metabolism has been previously demonstrated (Fig. 1D). Although no methanogens that encode Acs and Rnf, i.e., the category III strains, have been shown to grow on acetate as the sole carbon and energy substrate, we decided to pursue a more formal test of this hypothesis using *M. acetivorans*. We reasoned that this direct genetic approach was more appropriate than re-attempting cultivation experiments with category III strains, as there is a wealth of experimental data describing how *M. acetivorans* grows on acetate, allowing us to test the role of individual modules in the acetoclastic pathway.

To explore acetate metabolism via Acs and Rnf, we generated a ∆*ack-pta* strain of *M. acetivorans*, which cannot grow on acetate due to a disruption in the substrate activation pathway (Fig. 2). Our data validate previous reports that transposon insertions into these genes abrogate growth on acetate (28). However, when we attempted to complement our ∆*ack-pta* mutant with *acs* sequences from across the *Methanosarcinia*, these genes failed to rescue acetoclastic growth despite measurable acetyl-CoA production in cell lysates. We note reduced acetyl-CoA production in the *∆ack-pta/acs* mutants compared to the *∆ack-pta/ack-pta* complementation strain but interpret this outcome to be reflective of the lower V_max_ of Acs versus Ack, as has been previously reported (5, 20). Furthermore, reduced acetyl-CoA production would only slow down the growth of *∆ack-pta/acs* mutants compared to WT, which would be more in line with previous observations of slower growth rates among the *Methanothrix* spp. compared to the *Methanosarcina* spp. (5). The complete lack of growth in our media conditions suggests that the Acs and Rnf modules are functionally incompatible for acetoclastic growth in methanogens. Based on this outcome, we rule out the possibility of acetoclastic methanogenesis in category III methanogens but posit that Acs might be retained in these organisms for the purpose of supplementing biomass production during methylotrophic growth, which could be confirmed with future work in these strains. Future experiments could also explore growth at lower concentrations of acetate in our genetically-engineered category III strains, as Acs might provide an additional selective advantage under these conditions.

In parallel, we also addressed the likelihood of acetate growth in a putative class IV strain, i.e., one that would encode Ack+Pta and Fpo′. Although this combination of genes was not detected in any genome in our bioinformatic screen, Fpo′ is hypothesized to be the energy conservation module required for acetoclastic growth in *Methanothrix*, but this has not been directly tested due to the genetic intractability of *Methanothrix* spp. (15, 16) Additionally, mutants of *Methanosarcina mazei* lacking Ech can still generate a chemiosmotic gradient with Fd_red_, which is hypothesized to be mediated by the Fpo′ complex (29). Therefore, we used mutants of *M. acetivorans* that express the Fpo′ complex instead. Our results suggest that there is a fundamental incompatibility between Ack+Pta and Fpo′ as modules for mediating acetoclastic growth. One plausible reason is that the presence of FpoF in *M. acetivorans* precludes the rest of the Fpo complex, i.e., Fpo′, from interacting with Fd. Alternately, there might also be incompatibility between the Fd_red_ produced during acetate metabolism and the active site of the potential Fpo′ complex generated in our mutants, as ferredoxin specificity has been noted for other metabolic processes in bacteria and archaea (30–34). It should also be noted that despite encoding Acs and Fpo′, *Methermicoccus shengliensis* has not been reported to grow with acetate as the sole substrate (35, 36). Thus, the lack of acetoclastic growth in our ∆*mmcA-rnf* P*_mcrB_*_(tetO4)_-*fpo*′ strain might be due to additional proteins in *Methanothrix* spp. strains that modify the Fpo′ to interact with Fd, as has been observed for Fd-interacting complex I homologs in chloroplasts (37, 38). Future work on the *Methanothrix* Fpo′ complex will help to answer how this complex interacts with Fd.

**Fig 5:**
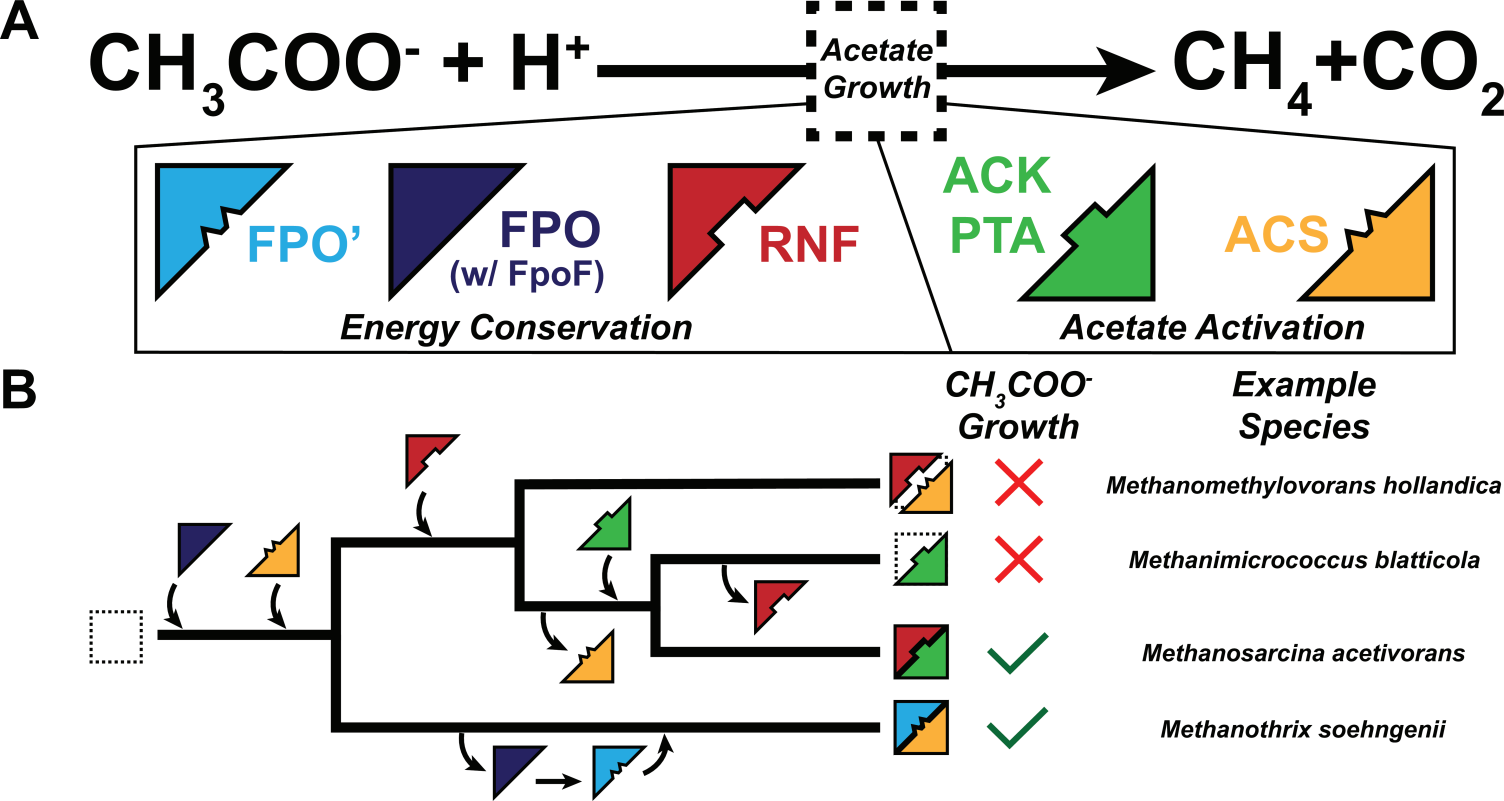
Co-evolution of energy conservation and acetate activation modules is necessary to enable acetoclastic methanogenesis. (**A**) Model depicting energy conservation and acetate activation modules as colored blocks that must interact precisely to fill in the empty square representing acetoclastic methanogenesis. (**B**) The provenance of enzyme modules from panel A among select methanogens within the class *Methanosarcinia*. An ancestral strain might have encoded Fpo (dark blue) along with Acs (yellow). However, to enable acetoclastic growth in the *Methanothrix*, Fpo would have had to undergo loss of FpoF and potentially further modification to generate Fpo′ (light blue) compatible with Acs for acetoclastic methanogenesis. In the other methanogens, Rnf (red) was likely acquired after the most recent common ancestor of category I diverged from the *Methanotrichales*. Methanogenic ancestors with Rnf were likely precluded from using acetate as a sole substrate until Acs was replaced with Ack+Pta (green) in the ancestor of *Methanosarcina* and *Methanomicrococcus*, which facilitated a new form of acetoclastic methanogenesis (category II). Loss of Rnf in *Methanomicrococcus* due to their adaptation to host-associated niches led to a loss of acetoclastic methanogenesis in this group of methanogens.

Our results point to incompatibility between modules of acetate activation and energy conservation that have not already been described in isolated acetoclastic methanogens (Fig. 5A) and therefore highlight an important nuance in the evolutionary history of acetoclastic methanogenesis within the *Methanosarcinia*. Previous hypotheses have implicated horizontal gene transfer as being the major driver of acetoclastic metabolism among Ack+Pta containing methanogens (6, 39), but our results emphasize that the horizontal acquisition of catabolic genes alone is not sufficient, and that integration with energy-conserving modules of the ETC would also be required. Thus, the transitions to acetoclastic metabolism that have occurred among the common ancestors of the extant *Methanosarcinia* have been shaped by coordinated evolutionary trajectories of the ETC among the *Methanothrix*, and the substrate activation genes among the *Methanosarcina* (Fig. 5B). Building on this study, future work is necessary for detailing the physiological and ecological adaptations that facilitate acetoclastic methanogenesis in *Methanosarcina* spp. and *Methanothrix* spp.

## MATERIALS AND METHODS

### Survey of genome taxonomy database

Genomes from GTDB r214.0 (https://data.gtdb.ecogenomic.org/releases/release214/214.0/) were downloaded and annotated with Prokka v.1.14.6 using the prokaryotic genetic code 11 (40). Genome accessions belonging to the class *Methanosarcinia* were determined using GTDB taxonomy metadata and the associated annotated genomes were selected for downstream genomic surveys. Note that the species we referred to as “*M. harundinacea*” in our analyses are currently being scrutinized for reclassification as “*Methanocrinis harundinaceus*” as proposed by Khomyakova et al. (41). However, since the reclassification has not been formalized, we chose to refer to these organisms using their previous designation within the “*Methanothrix*” genus. CheckM completeness scores were calculated using a concatenated biomarker set described in https://data.gtdb.ecogenomic.org/releases/release214/214.0/METHODS.txt (42). We obtained profile HMMs for acetate catabolism: acetate kinase (Ack), phosphotransacetylase (Pta), and AMP-forming acetyl-CoA synthetase (Acs), and respiratory complexes: the *Rhodobacter* nitrogen fixation complex (in methanogens MmcA-RnfCDGEAB), the Energy converting hydrogenase complex (EchABCDEF), and the F_420_:methanophenazine oxidoreductase (FpoABCDHIJKLMNOF) from the NCBI or KoalaFAM (43). A list of all accessions used is available in Table S8. We used a custom command line tool (https://github.com/kshalv/hmm_tools/tree/main) (44) to generate distributions for proteins involved in the activation of acetate to acetyl-CoA and energy conservation. Briefly, we used the “hmm” option of the command line tool to iterate through the given profile HMMs with HMMER 3.4 (hmmer.org [45]), and recorded hits with an e-value threshold lower than a 1E-03 threshold. In our initial analyses, we used the trusted cutoff (TC) score threshold for all queries. We noticed that genomes belonging to the family *Methanotrichaceae* were lacking hits for several of the subunits of the Fpo. We inspected complete genomes of *M. soehngenii* (NCBI accession: NC_015416.1) and *M. harundinacea* (NCBI accession: NC_017527.1) and were able to identify missing subunits manually. Thus, to capture the potential diversity of Fpo complexes across all *Methanosarcinia*, we ran our HMM analysis again using the noise cutoff (NC) score threshold for all profile HMMs. We found that the NC threshold recapitulated the TC threshold results for all proteins except Fpo, in which more subunits were identified among the *Methanotrichaceae*. Thus, we reasoned that the NC hit score prediction was accurate to cellular physiology. All protein hits that exceeded the NC threshold were counted and recorded for each genome to generate a presence/absence distribution table. The presence/absence hit distribution was overlaid on a tree of *Methanosarcinia* that was generated from the ar53_r214.tree available through GTDB. For energy conservation complexes composed of multiple subunits, a threshold number of hits had to be successfully detected for “presence” to be counted, which was ≥4 of six Rnf subunits; ≥5 of seven Fpo′ subunits; ≥4 of six Ech subunits.

### Media and culture conditions

*M. acetivorans* strains were grown in bicarbonate-buffered high-salt (HS) liquid medium containing 50 mM trimethylamine (TMA), 40 mM sodium acetate, or a combination of 50 mM TMA and 20 mM acetate as the growth substrate and an 80:20 mix of N_2_:CO_2_ gas in the headspace at approximately 55–69 kPa (46. All substrates were added prior to autoclaving. For Liquid cultures of *M. acetivorans* strains were incubated without shaking at 37°C. To generate mutants, cells were plated on 50 mM TMA with 1.5% wt/vol agar (Sigma-Aldrich, St. Louis, MO, USA) also containing 2 µg/mL Puromycin as a selective agent. Puromycin was added to agar-solidified HS-TMA after autoclaving from a 1,000× sterile, anaerobic stock solution with N_2_ gas in the headspace at approximately 55–69 kPa. Agar-solidified HS+TMA+Puromycin plates were incubated at 37°C in a custom-built intra-chamber anaerobic incubator with a headspace of 79.9:20:0.1 N_2_:CO_2_:H_2_S, as described previously (47). Where indicated, mutant strains were cultured with 2 µg/mL Puromycin to maintain plasmids and various amounts of tetracycline hydrochloride to induce expression of genes from tetracycline-inducible promoters as described previously (48). Puromycin and tetracycline were added to culture tubes after autoclaving but before inoculation from 10× or 100× filter-sterilized, anaerobic stock solutions with N_2_ gas in the headspace at 55–69 kPa.

*Escherichia coli* strains were routinely grown using lysogeny broth (LB). For liquid culturing, strains were incubated at 37°C at 250 rpm in a shaking incubator (Thermo Fisher Scientific, Waltham, MA, USA). For mutant generation, strains were plated on LB with 1.5% wt/vol agar with 25 µg/mL kanamycin and/or 10 µg/mL chloramphenicol. For plasmid extraction, strains were grown in liquid LB with equivalent concentrations of antibiotics as agar plates and 10 mM rhamnose.

### Plasmid construction

For CRISPR-Cas9-mediated gene editing, plasmids were constructed as described previously (49) using Gibson assembly (50). Briefly, 20 bp guide sequences were designed against the *M. acetivorans* C2A genome using the Find CRISPR Sites tool with an NGG 3′ PAM site using the Geneious platform (v.11.0). The 20-bp guides were synthesized as overhangs on primers (Integrated DNA Technologies, Coralville, IA, USA) used to introduce the sgRNA cassette into *AscI*-digested pDN201 (49). A list of sgRNA targeting sequences can be found in Table S9. In the same plasmid backbone at the *PmeI* site, a *ca*. 2 kbp homology-directed repair template was cloned to generate the edits of interest at the sites cut by the sgRNA cassette. For in-frame gene deletions, *ca*. 1 kb upstream and *ca*. 1 kb downstream of the gene(s) of interest were amplified from the chromosome, leaving only 30 bp of the 5′ and 3′ ends of the gene(s) in the repair template. For the promoter swap mutations, a commercial DNA fragment (Integrated DNA Technologies, Coralville, IA, USA) encoding the desired terminator and tetracycline-inducible P*_mcrB_*_(tetO4)_ promoter (~400 bp) was fused with *ca*. 850 bp upstream and *ca*. 730 bp downstream homology arms amplified via PCR from the chromosome. Complementation plasmids were also generated via Gibson assembly (50) by cloning in the gene(s) of interest either from commercial gene fragments (Twist Bioscience, South San Francisco, CA, USA) or from genes amplified via PCR from the chromosome of *M. acetivorans* or *M. hollandica* under a tetracycline-inducible P*_mcrB_*_(tetO1)_ promoter in pJK027A (48). The pDN201- and pJK027A-derived plasmids were retrofitted with pAMG40 for autonomous replication in *M. acetivorans* using Invitrogen Gateway BP Clonase II Enzyme mix (Thermo Fisher Scientific, Waltham, MA, USA) as previously described (48). Plasmids were transformed into *E. coli* strain WM4489 by electroporation (MicroPulser Electroporator, Bio-Rad, Hercules, CA, USA). Plasmids were extracted from host *E. coli* strains using the Zymo Zyppy Plasmid Miniprep Kit (Zymo Research, Irvine, CA, USA). Plasmids were confirmed using PCR and Sanger sequencing (Barker Sequencing Facility, UC Berkeley, Berkeley, CA, USA) or restriction endonuclease digestion. Plasmids used for mutant generation are listed in Table S10. A complete list of primers used in this study is available in Table S11.

### *M. acetivorans* mutant generation

Mutants of *M. acetivorans* were generated using a liposome-mediated transformation protocol inside an anaerobic chamber with a gas atmosphere of 78:18:4 N_2_:CO_2_:H_2_ as previously described (51). In brief, 20 mL of late exponential phase cultures (OD600 ~ 0.8–1.0) were harvested by centrifugation in the anaerobic chamber. The cell pellet was resuspended in 750 µL of anaerobic, isotonic, bicarbonate-buffered sucrose (pH = 7.4) containing 100 µM cysteine. Next, 25 μL of N-[1-(2,3-dioleoyloxy)propyl]-N,N,N-trimethylammonium methylsulfate (DOTAP, Roche Diagnostics Deutschland GmbH, Mannheim, Germany) or 3 µL of DOTAP chloride (MedChemExpress, Monmouth Junction, NJ, USA) and 2 μg of plasmid DNA resuspended in 50 µL of sucrose buffer were pre-incubated with 75 µL (or 97 µL) of anaerobic buffered sucrose for 30 min before being added to the cell suspension mixture. Transfections with the DOTAP+DNA liposomes and cells were incubated at room temperature for 4 h in the anaerobic chamber before inoculation into 10 mL of 50 mM HS-TMA. Outgrowths of transfected cells were incubated at 37°C for 12–16 h before plating on 50 mM HS-TMA agar solidified medium with 2 µg/mL Puromycin as a selective agent. For clearing the CRISPR-Cas9-containing plasmids from deletion mutants, strains were plated on 50 mM HS-TMA agar with 20–160 µg/mL 8-aza-diaminopurine (8ADP). Strains were genotyped for clearance of the mutagenic plasmid by diagnostic PCR for *pac*, the Puromycin resistance gene, and/or whole-genome sequencing. A full list of strains used in this study is available in Table 3.

**TABLE 3:** *M. acetivorans* strains used in this study

Strain	Genotype	Construction details	Source
WWM60	∆*hpt*::P*_mcrB_-tetR*	–^*b*^	Guss et al. (48)
WWM1015	∆*hpt*::P*_mcrB_*-*phiC31int-attB* ∆*mmcA-rnf^a^*	–	Mand (52)
DDN227	∆*hpt*::P*_mcrB_-tetR* ∆*mreA*	WWM60 was transformed to Pur^R^ with pBD014; plasmid-cured strain was isolated by plating on medium with 8ADP	This study
DDN230	∆*hpt*::P*_mcrB_-phiC31int-attB* ∆*mmcA-rnf* ∆*mreA*	WWM1015 was transformed to Pur^R^ with pBD014; plasmid-cured strain was isolated by plating on medium with 8ADP	This study
DDN235	∆*hpt*::P*_mcrB_-tetR* ∆*ack-pta*	WWM60 was transformed to Pur^R^ with pBD024; plasmid-cured strain was isolated by plating on medium with 8ADP	This study
DDN264	∆*hpt*::P*_mcrB_-tetR* T*_mcr_* P*_mcrB_*_(tetO4)_*-fpo*	WWM60 was transformed to Pur^R^ with pBD030; plasmid-cured strain was isolated by plating on medium with 8ADP	This study
DDN306	∆*hpt*::P*_mcrB_-phiC31int-attB* ∆*mmcA-rnf* T*_mcr_* P*_mcrB_*_(tetO4)_*-fpo*	WWM60 was transformed to Pur^R^ with pBD030; plasmid-cured strain was isolated by plating on medium with 8ADP	This study
DDN345	WWM60/pBD035 [P*_mcrB_*_(tetO1)_*-acs3*_MTS_]	WWM60 was transformed to Pur^R^ with pBD035; isolates were verified by Sanger sequencing and grown in HS medium supplemented with 2 µg/mL Puromycin	This study
DDN347	WWM60/pBD044 [P*_mcrB_*_(tetO1)_*-acs3*_MMH_]	WWM60 was transformed to Pur^R^ with pBD044; isolates were verified by Sanger sequencing and grown in HS medium supplemented with 2 µg/mL Puromycin	This study
DDN348	DDN235/pBD043 [P*_mcrB_*_(tetO1)_*-ack-pta*]	DDN235 was transformed to Pur^R^ with pBD043; isolates were verified by Sanger sequencing and grown in HS medium supplemented with 2 µg/mL Puromycin	This study
DDN349	DDN235/pBD044 [P*_mcrB_*_(tetO1)_*-acs3*_MMH_]	DDN235 was transformed to Pur^R^ with pBD044; isolates were verified by Sanger sequencing and grown in HS medium supplemented with 2 µg/mL Puromycin	This study
DDN350	WWM60/pBD037 [P*_mcrB_*_(tetO1)_*-acs3*_MTH_]	WWM60 was transformed to Pur^R^ with pBD037; isolates were verified by Sanger sequencing and grown in HS medium supplemented with 2 µg/mL Puromycin	This study
DDN352	DDN235/pBD035 [P*_mcrB_*_(tetO1)_*-acs3*_MTS_]	DDN235 was transformed to Pur^R^ with pBD035; isolates were verified by Sanger sequencing and grown in HS medium supplemented with 2 µg/mL Puromycin	This study
DDN353	DDN235/pBD037 [P*_mcrB_*_(tetO1)_*-acs3*_MTH_]	DDN235 was transformed to Pur^R^ with pBD037; isolates were verified by Sanger sequencing and grown in HS medium supplemented with 2 µg/mL Puromycin	This study
DDN354	DDN235/pBD038 [P*_mcrB_*_(tetO1)_*-acs3*_MTH_+*PPase*_MTH_]	DDN235 was transformed to Pur^R^ with pBD038; isolates were verified by Sanger sequencing and grown in HS medium supplemented with 2 µg/mL Puromycin	This study
DDN360	WWM60/pBD036 [P*_mcrB_*_(tetO1)_*-acs3*_MTS_+*PPase*_MTS_]	WWM60 was transformed to Pur^R^ with pBD036; isolates were verified by Sanger sequencing and grown in HS medium supplemented with 2 µg/mL Puromycin	This study
DDN361	WWM60/pBD038 [P*_mcrB_*_(tetO1)_-*acs3*_MTH_+*PPase*_MTH_]	WWM60 was transformed to Pur^R^ with pBD038; isolates were verified by Sanger sequencing and grown in HS medium supplemented with 2 µg/mL Puromycin	This study
DDN362	DDN235/pBD036 [P*_mcrB_*_(tetO1)_*-acs3*_MTS_+*PPase*_MTS_]	DDN235 was transformed to Pur^R^ with pBD036; isolates were verified by Sanger sequencing and grown in HS medium supplemented with 2 µg/mL Puromycin	This study
DDN448	DDN235/pBD047 [P*_mcrB_*_MM_-*cdh2*, P*_mcrB_*_(tetO1)_-*acs3*_MTH_*+PPase*_MTH_]	DDN235 transformed to PurR with pBD047; isolates were verified by Sanger sequencing and grown in HS medium supplemented with 2 µg/mL Puromycin	This study

^*a*^ Sequencing results showed that this strain contains the phiC31 attB rather than attP as previously stated.

^*b*^ – indicates that construction details are not described.

### Genomic DNA extraction, sequencing, and analysis

Approximately 2 mL of cells were harvested from a saturated culture of *M. acetivorans* and genomic DNA was extracted using the Qiagen DNeasy Blood & Tissue kit (Qiagen, Hilden, Germany) according to the manufacturer’s instructions. Samples were sent for library preparation and whole-genome sequencing at SeqCenter (Pittsburgh, PA, USA). Analysis of the sequencing results was conducted using *breseq* v0.35.5 (53).

### Acetylhydroxamate assay for measuring acetyl-CoA production

A colorimetric assay to detect the production of acetyl groups from acetate in cleared cell lysates was developed based on previous methods (20, 21). Briefly, ~30 mL of TMA-grown or ~250–500 mL of acetate-grown *M. acetivorans* strains were harvested by centrifugation at 5,000 RPM at 4°C for 15 min (Sorvall Legend XTR,472 Thermo Fisher Scientific, Waltham, MA, USA). Cell pellets were lysed using 600 µL of 50 mM sodium phosphate buffer (pH = 8.0) on ice. A volume of 1–2 µL of DNase was added and the lysate was incubated at room temperature for 10 min to digest DNA. After incubation, 36 µL of 5 M sodium chloride was added to the lysates and incubated for 2 min at room temperature. The cell lysate mixture was clarified by centrifugation at 14,000 RPM at 4°C for 10 min (Sorvall Legend XTR,472 Thermo Fisher Scientific, Waltham, MA, USA). This cleared lysate was used for assays. Total protein concentration was measured in a microplate reader (BioTek Epoch 2, Winooski, VT, USA) by adding 5 µL of cleared lysate to 250 µL of Pierce Bradford Plus Protein Assay Reagent (Thermo Fisher Scientific, Waltham, MA, USA) and calibrating against a standard curve of BSA (54). The assay buffer contained 100 mM Tris.HCl (pH = 8.5), 10 mM sodium acetate, 0.2 mM coenzyme A, 4 mM MgCl_2_, 2 mM adenosine 5′triphosphate disodium salt (ATP), 2 mM dithiothreitol, and 600 mM dilute, neutralized hydroxylamine. Dilute, neutralized hydroxylamine was prepared by combining equal parts of 4 M hydroxylamine HCl and 14.7% wt/vol potassium hydroxide, which was then diluted 1:10 with MilliQ water. Reactions were started by adding 1 mg of cleared lysate to 333 µL of reaction buffer, and samples were incubated at 37°C for 30 min in technical duplicate or triplicate. Reactions were quenched by bringing the total reaction volume to 666 µL using 10% trichloroacetic acid to precipitate protein. Finally, 333 µL of 2.5% wt/vol Fe(III)Cl_3_ in 2 M HCl was added to each reaction to develop the color. Samples were measured for absorbance at 540 nm in 10 × 4 × 45 mm plastic cuvettes using a UV-Vis spectrophotometer (Genesys 50, Thermo Fisher Scientific, Waltham, MA, USA) with cuvette attachment. Absorbance of experimental samples was blanked against a no-cell control reaction containing 333 µL of reaction buffer, 333 µL of 10% trichloroacetic acid, and 333 µL of Fe(III)Cl_3_. An acetyl-CoA standard curve was generated by measuring absorbance at 540 nm of cell-free reactions that contained a final concentration of 0.25, 0.5, 1, 2.5, 5, 7.5, 10, and 20 mM of acetyl-CoA (Cayman Chemical, Ann Arbor, MI, USA).

### Growth assays

For growth analysis, cultures of *M. acetivorans* strains were inoculated into 26 mL Balch tubes containing 10 mL of fresh media, and growth was monitored as the increase in absorbance at 600 nm over time (optical density, OD600) using a UV-Vis spectrophotometer (Genesys 50, Thermo Fisher Scientific, Waltham, MA, USA). Triplicate or quadruplicate replicate tubes were used for experiments as indicated. All strains were pre-cultured in 50 mM TMA before transfer for growth analysis. For 50 mM TMA growth curves, a ~1:20 dilution of inoculum from an early stationary phase culture was used (~0.5 mL into 10 mL fresh medium). For 40 mM acetate growth curves, an ~1:11 dilution of inoculum from an early stationary phase culture was used (~1 mL into 10 mL fresh medium). Growth rates and doubling times (T_D_) were calculated from the slopes of log10-transformed OD600 values from the exponential phase by linear regression analysis with R^2^ values ≥0.95. Lag/acclimation times (T_Lag_) were calculated as the time in hours where the slope of the linear regression intersected with the log10-transformed OD600 value from t = 0 h (y-intercept). Analysis of growth data and statistical tests was performed in Microsoft Excel (v16.96.1). Growth curves were plotted using GraphPad Prism (v10.4.2).

### RNA extraction, sequencing, and analysis

Three replicate 11 mL cultures of *M. acetivorans* strains growing on 40 mM acetate were harvested and lysed at mid-exponential phase (OD600 ~0.100) by 1:1 addition (11 mL) of Trizol reagent (Life Technologies, Carlsbad, CA, USA) and incubated at room temperature for 5 min. To this mixture, 22 mL of 100% ethanol was added. RNA extraction was performed using the Qiagen RNEasy Mini Kit (Qiagen, Hilden, Germany) according to the manufacturer’s instructions. Quantification of the RNA yield was performed using a Nanodrop One/OneC UV Spectrophotometer (Thermo Fisher Scientific, Waltham, MA, USA) before storage at −80°C. Samples were shipped on dry ice to SeqCenter (Pittsburgh, PA, USA) for rRNA depletion, library preparation, and Illumina sequencing. Non-interleaved, paired-end reads were uploaded to KBase, as previously described (12, 55). Reads were aligned to the *M. acetivorans* C2A genome using Bowtie (v2.3.2), assembled using Cufflinks (v.2.2.1), and differential expression analysis was performed using DESeq2 (v1.20.0). Changes in transcript abundance were determined to be “significant” if the q-value ≤0.01. A volcano plot for global transcriptome response and bar graphs for individual gene expression changes was generated using GraphPad Prism (v10.4.2).

## Data Availability

All sequencing data have been deposited in the NCBI Sequence Read Archive under BioProject number PRJNA1365152.

## SUPPLEMENTAL MATERIAL

Supplemental figures jb.00448-25-s0001.docx Figures S1 to S6.

Supplemental tables jb.00448-25-s0002.xlsx Tables S1 to S11.

ASM does not own the copyrights to Supplemental Material that may be linked to, or accessed through, an article. The authors have granted ASM a non-exclusive, world-wide license to publish the Supplemental Material files. Please contact the corresponding author directly for reuse.

## References

[1] Dean JF, Middelburg JJ, Röckmann T, Aerts R, Blauw LG, Egger M, Jetten MSM, de Jong AEE, Meisel OH, Rasigraf O, Slomp CP, in’t Zandt MH, Dolman AJ. 2018. Methane feedbacks to the global climate system in a warmer world. Rev Geophys 56:207–250. doi:10.1002/2017RG000559

[2] Jackson RB, Saunois M, Martinez A, Canadell JG, Yu X, Li M, Poulter B, Raymond PA, Regnier P, Ciais P, Davis SJ, Patra PK. 2024. Human activities now fuel two-thirds of global methane emissions. Environ Res Lett 19:101002. doi:10.1088/1748-9326/ad6463

[3] Thauer RK, Kaster A-K, Seedorf H, Buckel W, Hedderich R. 2008. Methanogenic archaea: ecologically relevant differences in energy conservation. Nat Rev Microbiol 6:579–591. doi:10.1038/nrmicro1931 18587410

[4] Thauer RK. 1998. Biochemistry of methanogenesis: a tribute to marjory stephenson:1998 marjory stephenson prize lecture. Microbiology (Reading, Engl) 144:2377–2406. doi:10.1099/00221287-144-9-2377 9782487

[5] Jetten MSM, Stams AJM, Zehnder AJB. 1992. Methanogenesis from acetate: a comparison of the acetate metabolism in Methanothrix soehngenii and Methanosarcina spp. FEMS Microbiol Lett 88:181–197. doi:10.1016/0378-1097(92)90802-U

[6] Fournier GP, Gogarten JP. 2008. Evolution of acetoclastic methanogenesis in Methanosarcina via horizontal gene transfer from cellulolytic Clostridia. J Bacteriol 190:1124–1127. doi:10.1128/JB.01382-07 18055595 PMC2223567

[7] Ferry JG. 1992. Methane from acetate. J Bacteriol 174:5489–5495. doi:10.1128/jb.174.17.5489-5495.1992 1512186 PMC206491

[8] Jetten MSM, Fluit TJ, Stams AJM, Zehnder AJB. 1992. A fluoride-insensitive inorganic pyrophosphatase isolated from Methanothrix soehngenii. Arch Microbiol 157:284–289. doi:10.1007/BF00245163 1324658

[9] Downing BE, Nayak DD. 2025. Innovations in the electron transport chain fuel archaeal methane metabolism. Trends Biochem Sci 50:425–437. doi:10.1016/j.tibs.2025.02.004 40133173

[10] Hedderich R, Forzi L. 2005. Energy-converting [NiFe] hydrogenases: more than just H2 activation. Microb Physiol 10:92–104. doi:10.1159/000091557 16645307

[11] Schlegel K, Welte C, Deppenmeier U, Müller V. 2012. Electron transport during aceticlastic methanogenesis by Methanosarcina acetivorans involves a sodium-translocating Rnf complex. FEBS J 279:4444–4452. doi:10.1111/febs.12031 23066798

[12] Downing BE, Gupta D, Nayak DD. 2023. The dual role of a multi-heme cytochrome in methanogenesis: MmcA is important for energy conservation and carbon metabolism in Methanosarcina acetivorans. Mol Microbiol 119:350–363. doi:10.1111/mmi.15029 36660820

[13] Mand TD, Metcalf WW. 2019. Energy conservation and hydrogenase function in methanogenic archaea, in particular the genus Methanosarcina Microbiol Mol Biol Rev 83:e00020-19. doi:10.1128/MMBR.00020-19 31533962 PMC6759668

[14] Welte C, Krätzer C, Deppenmeier U. 2010. Involvement of Ech hydrogenase in energy conservation of Methanosarcina mazei: function of Ech in energy conservation. FEBS J 277:3396–3403. doi:10.1111/j.1742-4658.2010.07744.x 20629748

[15] Welte C, Deppenmeier U. 2014. Bioenergetics and anaerobic respiratory chains of aceticlastic methanogens. Biochimica et Biophysica Acta (BBA) - Bioenergetics 1837:1130–1147. doi:10.1016/j.bbabio.2013.12.002 24333786

[16] Welte C, Deppenmeier U. 2011. Membrane-bound electron transport in Methanosaeta thermophila. J Bacteriol 193:2868–2870. doi:10.1128/JB.00162-11 21478356 PMC3133127

[17] Smith KS, Ingram-Smith C. 2007. Methanosaeta, the forgotten methanogen? Trends Microbiol 15:150–155. doi:10.1016/j.tim.2007.02.002 17320399

[18] Thomas CM, Taib N, Gribaldo S, Borrel G. 2021. Comparative genomic analysis of Methanimicrococcus blatticola provides insights into host adaptation in archaea and the evolution of methanogenesis. ISME Commun 1:47. doi:10.1038/s43705-021-00050-y 37938279 PMC9723798

[19] Baumer S, Ide T, Jacobi C, Johann A, Gottschalk G, Deppenmeier U. 2000. The F420H2 dehydrogenase from Methanosarcina mazei is a Redox-driven proton pump closely related to NADH dehydrogenases. J Biol Chem 275:17968–17973. doi:10.1074/jbc.M000650200 10751389

[20] Jetten MSM, Stams AJM, Zehnder AJB. 1989. Isolation and characterization of acetyl-coenzyme A synthetase from Methanothrix soehngenii. J Bacteriol 171:5430–5435. doi:10.1128/jb.171.10.5430-5435.1989 2571608 PMC210380

[21] Aceti DJ, Ferry JG. 1988. Purification and characterization of acetate kinase from acetate-grown Methanosarcina thermophila. Evidence for regulation of synthesis. J Biol Chem 263:15444–15448. 2844814

[22] Meng Y. 2010. Investigation of biochemistry and enzymology of acyl-coenzyme a synthetase, All Dissertations

[23] Zhu J, Zheng H, Ai G, Zhang G, Liu D, Liu X, Dong X. 2012. The genome characteristics and predicted function of methyl-group oxidation pathway in the obligate aceticlastic methanogens, Methanosaeta spp. PLoS One 7:e36756. doi:10.1371/journal.pone.0036756 22590603 PMC3349665

[24] Matschiavelli N, Oelgeschläger E, Cocchiararo B, Finke J, Rother M. 2012. Function and regulation of isoforms of carbon monoxide dehydrogenase/acetyl coenzyme A synthase in Methanosarcina acetivorans. J Bacteriol 194:5377–5387. doi:10.1128/JB.00881-12 22865842 PMC3457241

[25] Reichlen MJ, Vepachedu VR, Murakami KS, Ferry JG. 2012. MreA functions in the global regulation of methanogenic pathways in Methanosarcina acetivorans. mBio 3:e00189-12. doi:10.1128/mBio.00189-12 22851658 PMC3419521

[26] Monod J. 1949. The growth of bacterial cultures. Annu Rev Microbiol 3:371–394. doi:10.1146/annurev.mi.03.100149.002103

[27] Chadwick GL, Dury GA, Nayak DD. 2024. Physiological and transcriptomic response to methyl-coenzyme M reductase limitation in Methanosarcina acetivorans. Appl Environ Microbiol 90:e0222023. doi:10.1128/aem.02220-23 38916294 PMC11267899

[28] Rother M, Metcalf WW. 2004. Anaerobic growth of Methanosarcina acetivorans C2A on carbon monoxide: an unusual way of life for a methanogenic archaeon. Proc Natl Acad Sci USA 101:16929–16934. doi:10.1073/pnas.0407486101 15550538 PMC529327

[29] Welte C, Kallnik V, Grapp M, Bender G, Ragsdale S, Deppenmeier U. 2010. Function of Ech hydrogenase in ferredoxin-dependent, membrane-bound electron transport in Methanosarcina mazei. J Bacteriol 192:674–678. doi:10.1128/JB.01307-09 19948802 PMC2812462

[30] Fixen KR, Pal Chowdhury N, Martinez‐Perez M, Poudel S, Boyd ES, Harwood CS. 2018. The path of electron transfer to nitrogenase in a phototrophic alpha‐proteobacterium. Environ Microbiol 20:2500–2508. doi:10.1111/1462-2920.14262 29708646

[31] Lewis NM, Sarne A, Fixen KR. 2023. Evolving a new electron transfer pathway for nitrogen fixation uncovers an electron bifurcating-like enzyme involved in anaerobic aromatic compound degradation. mBio 14:e0288122. doi:10.1128/mbio.02881-22 36645294 PMC9973337

[32] Jiménez-Vicente E, Navarro-Rodríguez M, Poza-Carrión C, Rubio LM. 2014. Role of Azotobacter vinelandii FdxN in FeMo-co biosynthesis. FEBS Lett 588:512–516. doi:10.1016/j.febslet.2013.12.018 24374338

[33] Addison H, Glatter T, K A Hochberg G, Rebelein JG. 2024. Two distinct ferredoxins are essential for nitrogen fixation by the iron nitrogenase in Rhodobacter capsulatus. mBio 15:e0331423. doi:10.1128/mbio.03314-23 38377621 PMC10936413

[34] Burkhart BW, Febvre HP, Santangelo TJ. 2019. Distinct physiological roles of the three ferredoxins encoded in the hyperthermophilic archaeon Thermococcus kodakarensis. mBio 10:02807–02818. doi:10.1128/mBio.02807-18 PMC6401487 30837343

[35] Mayumi D, Mochimaru H, Tamaki H, Yamamoto K, Yoshioka H, Suzuki Y, Kamagata Y, Sakata S. 2016. Methane production from coal by a single methanogen. Science 354:222–225. doi:10.1126/science.aaf8821 27738170

[36] Kurth JM, Nobu MK, Tamaki H, de Jonge N, Berger S, Jetten MSM, Yamamoto K, Mayumi D, Sakata S, Bai L, Cheng L, Nielsen JL, Kamagata Y, Wagner T, Welte CU. 2021. Methanogenic archaea use a bacteria-like methyltransferase system to demethoxylate aromatic compounds. ISME J 15:3549–3565. doi:10.1038/s41396-021-01025-6 34145392 PMC8630106

[37] Yamamoto H, Peng L, Fukao Y, Shikanai T. 2011. An Src homology 3 domain-like fold protein forms a ferredoxin binding site for the chloroplast NADH dehydrogenase-like complex in Arabidopsis. Plant Cell 23:1480–1493. doi:10.1105/tpc.110.080291 21505067 PMC3101538

[38] Friedrich T, Steinmüller K, Weiss H. 1995. The proton-pumping respiratory complex I of bacteria and mitochondria and its homologue in chloroplasts. FEBS Lett 367:107–111. doi:10.1016/0014-5793(95)00548-n 7796904

[39] Rothman DH, Fournier GP, French KL, Alm EJ, Boyle EA, Cao C, Summons RE. 2014. Methanogenic burst in the end-Permian carbon cycle. Proc Natl Acad Sci USA 111:5462–5467. doi:10.1073/pnas.1318106111 24706773 PMC3992638

[40] Seemann T. 2014. Prokka: rapid prokaryotic genome annotation. Bioinformatics 30:2068–2069. doi:10.1093/bioinformatics/btu153 24642063

[41] Khomyakova MA, Merkel AY, Slobodkin AI, Sorokin DY. 2023. Phenotypic and genomic characterization of the first alkaliphilic aceticlastic methanogens and proposal of a novel genus Methanocrinis gen.nov. within the family Methanotrichaceae Front Microbiol 14:1233691. doi:10.3389/fmicb.2023.1233691 37886072 PMC10598746

[42] Parks DH, Imelfort M, Skennerton CT, Hugenholtz P, Tyson GW. 2015. CheckM: assessing the quality of microbial genomes recovered from isolates, single cells, and metagenomes. Genome Res 25:1043–1055. doi:10.1101/gr.186072.114 25977477 PMC4484387

[43] Kanehisa M, Sato Y, Kawashima M, Furumichi M, Tanabe M. 2016. KEGG as a reference resource for gene and protein annotation. Nucleic Acids Res 44:D457–62. doi:10.1093/nar/gkv1070 26476454 PMC4702792

[44] Chadwick GL, Williams MC, Shalvarjian KE, Nayak DD. 2025. Methanogenesis marker 16 metalloprotein is the primary coenzyme M synthase in Methanosarcina acetivorans. PLoS Genet 21:e1011695. doi:10.1371/journal.pgen.1011695 40315266 PMC12068725

[45] Eddy SR. 2011. Accelerated profile HMM searches. PLoS Comput Biol 7:e1002195. doi:10.1371/journal.pcbi.1002195 22039361 PMC3197634

[46] Sowers KR, Boone JE, Gunsalus RP. 1993. Disaggregation of Methanosarcina spp. and growth as single cells at elevated osmolarity. Appl Environ Microbiol 59:3832–3839. doi:10.1128/aem.59.11.3832-3839.1993 16349092 PMC182538

[47] Metcalf WW, Zhang JK, Wolfe RS. 1998. An anaerobic, intrachamber incubator for growth of Methanosarcina spp. on methanol-containing solid media. Appl Environ Microbiol 64:768–770. doi:10.1128/AEM.64.2.768-770.1998 9464421 PMC106116

[48] Guss AM, Rother M, Zhang JK, Kulkarni G, Metcalf WW. 2008. New methods for tightly regulated gene expression and highly efficient chromosomal integration of cloned genes for Methanosarcina species. Archaea 2:193–203. doi:10.1155/2008/534081 19054746 PMC2685592

[49] Nayak DD, Metcalf WW. 2017. Cas9-mediated genome editing in the methanogenic archaeon Methanosarcina acetivorans. Proc Natl Acad Sci USA 114:2976–2981. doi:10.1073/pnas.1618596114 28265068 PMC5358397

[50] Thomas S, Maynard ND, Gill J. 2015. DNA library construction using Gibson Assembly. Nat Methods 12:i–ii. doi:10.1038/nmeth.f.384

[51] Metcalf WW, Zhang JK, Apolinario E, Sowers KR, Wolfe RS. 1997. A genetic system for Archaea of the genus Methanosarcina: liposome-mediated transformation and construction of shuttle vectors. Proc Natl Acad Sci USA 94:2626–2631. doi:10.1073/pnas.94.6.2626 9122246 PMC20139

[52] Mand T. 2018. Hydrogenase utilization and regulation in species of Methanosarcina PhD thesis, University of Illinois at Urbana–Champaign. https://hdl.handle.net/2142/102408.

[53] Deatherage DE, Barrick JE. 2014. Identification of Mutations in Laboratory-Evolved Microbes from Next-Generation Sequencing Data Using breseq, p 165–188. In Sun L, Shou W (ed), Engineering and Analyzing Multicellular Systems: Methods and Protocols. Springer, New York, NY. 10.1007/978-1-4939-0554-6_12 PMC4239701 24838886

[54] 2025. Bradford protein assay. Available from: https://bio-protocol.org/exchange/protocoldetail?id=45&type=1

[55] Arkin AP, Cottingham RW, Henry CS, Harris NL, Stevens RL, Maslov S, Dehal P, Ware D, Perez F, Canon S, et al.. 2018. KBase: the United States department of energy systems biology knowledgebase. Nat Biotechnol 36:566–569. doi:10.1038/nbt.4163 29979655 PMC6870991

