# Supplementary material for: Genus-specific remodeling of carbon and energy metabolism facilitates acetoclastic methanogenesis in Methanosarcina spp. and Methanothrix spp
Source: J Bacteriol. 2026 Jan 22;208(2):e00448-25. doi: 10.1128/jb.00448-25 (PMC12918732; doi:10.1128/jb.00448-25)
Supplement: Supplemental figures — Figures S1 to S6. [file jb.00448-25-s0001.docx]

**SUPPLEMENTARY FIGURES**


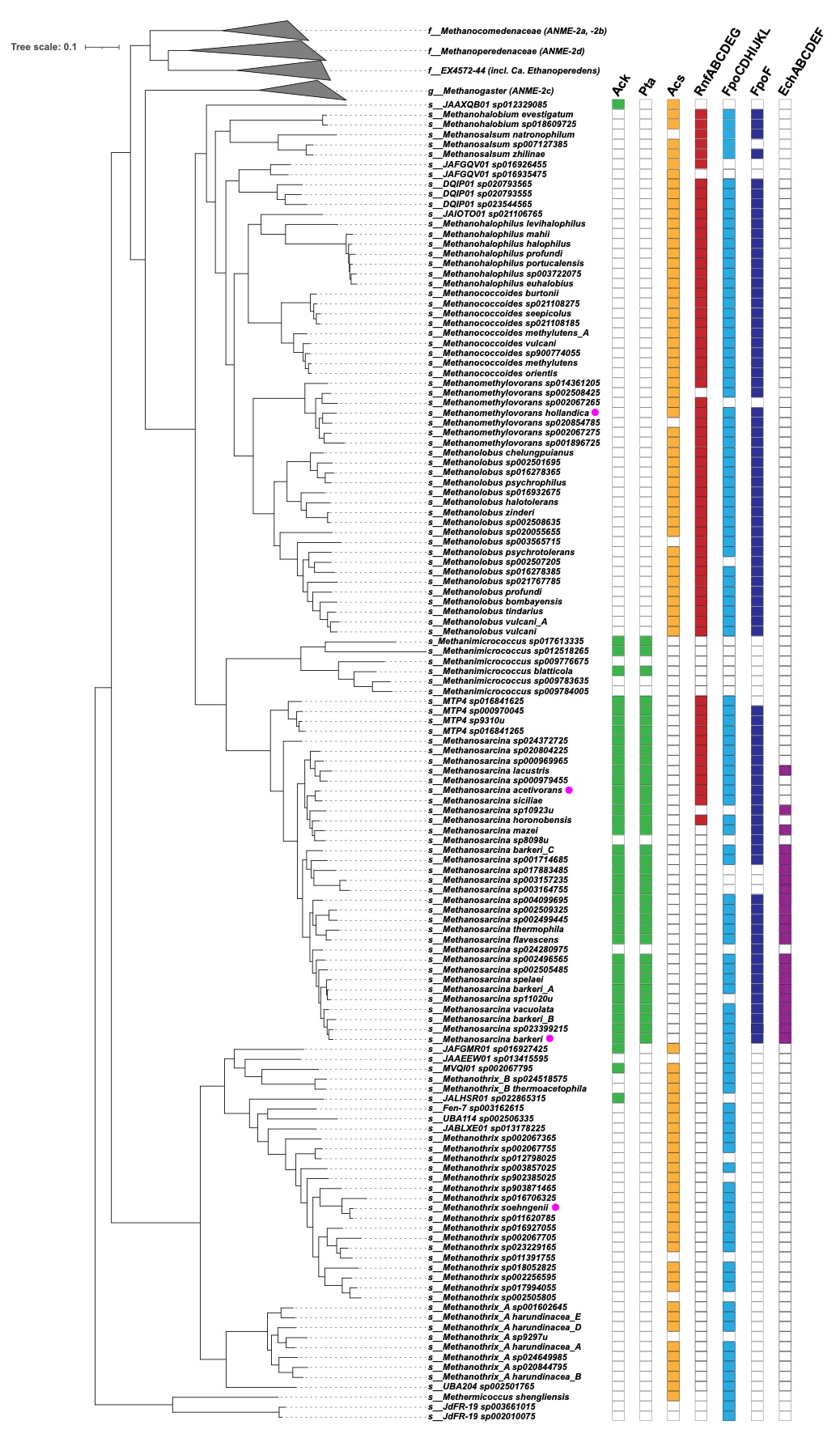


**Figure S1**. Comparative genomic analysis of acetate activation and electron transport chain genes across the class *Methanosarcinia*. For each species within the *Methanosarcinia* (GTDB r214.0), the presence or absence of the genes is indicated by a color-filled or empty box, respectively: Ack + Pta (acetate kinase + phosphotransacetylase), green; Acs [acetyl-CoA synthetase (AMP-forming)], yellow; Fpo’ (F_420_:phenazine oxidoreductase lacking FpoF subunit), light blue; FpoF (coenzyme F_420_ active site-containing subunit of F_420_:phenazine oxidoreductase), dark blue; Rnf (*Rhodobacter* nitrogen fixation complex), red; Ech (energy converting hydrogenase), purple. For energy conservation complexes composed of multiple subunits, a threshold number of hits had to be successfully detected for “presence” to be counted: For Rnf, ≥ 4 of 6 subunits; for Fpo’, ≥ 5 of 7 subunits; for Ech, ≥ 4 of 6 subunits. The four genomes represented in Figure 1B are indicated by pink circles.

**Figure S2.** The ∆*ack-pta* mutant phenocopies the parent strain in growth medium with trimethylamine (TMA) and acetate. Growth curve of WWM60 (WT; dark green circles) and the *∆ack-pta* mutant (white circles) in high-salt minimal medium containing 50 mM TMA and 20 mM acetate. Four replicate tubes of each strain used for the growth assays. Doubling times are not significantly different between strains (**Table S3**).


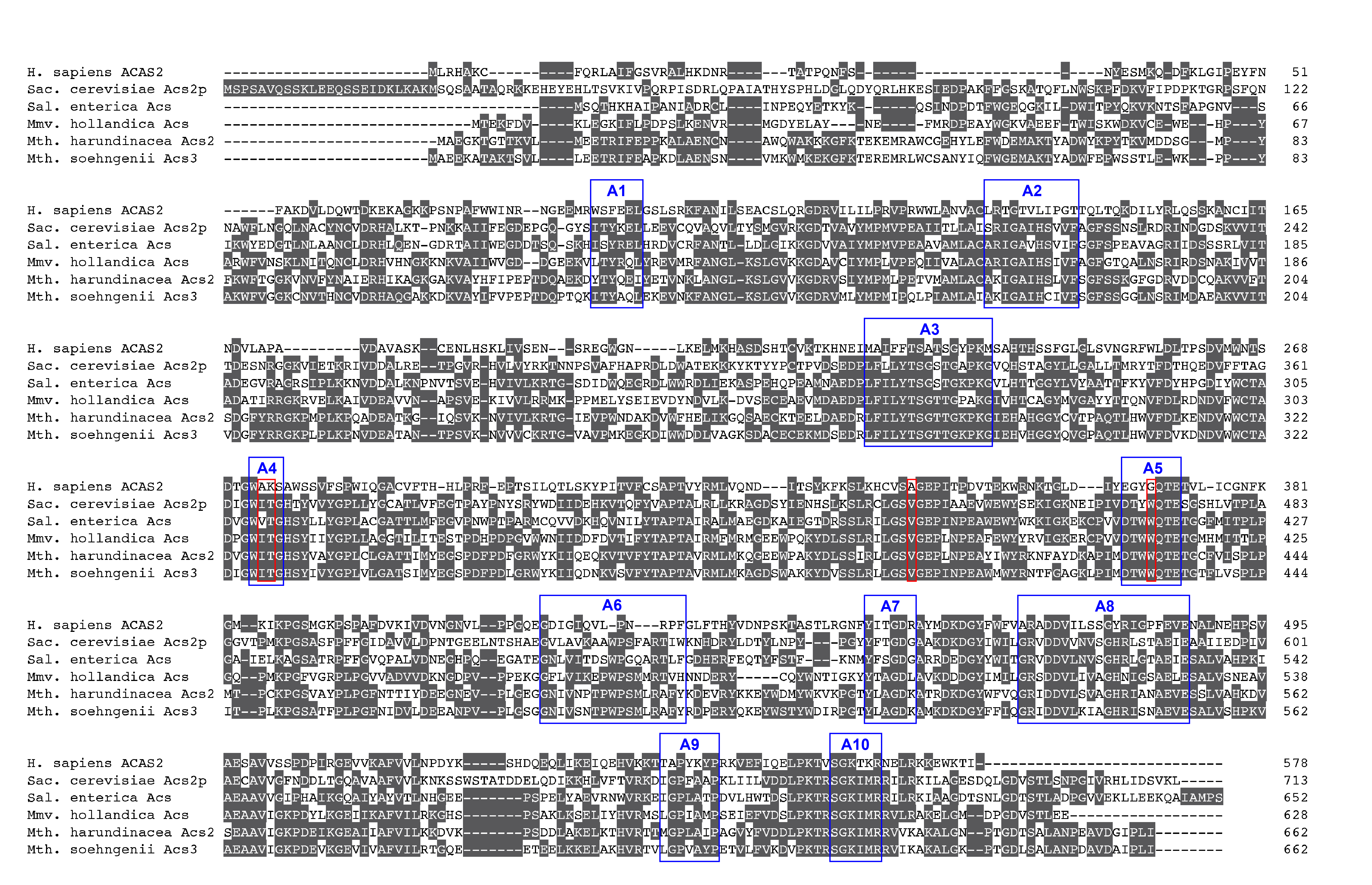


**Figure S3**. Multiple sequence alignment of acetyl-CoA synthetase (Acs) amino acid sequences from a variety of eukaryotic and prokaryotic organisms, including the three methanogen Acs sequences used to complement ∆*ack-pta* mutants of *Methanosarcina acetivorans* in this study. The alignment was performed using the Clustal Omega tool available through the Geneious platform (v.11.0). White letters on a gray background indicate consensus positions among the sequences used in this alignment. Black letters on a white background indicate non-consensus positions. The ten conserved motifs (A1-A10) highlighted in blue boxes belong to all AMP-forming acyl-CoA synthetase family proteins. The specific positions highlighted with red boxes refer to the predicted acetate-binding residues described by Meng (2010). Species abbreviations: H. sapiens, *Homo sapiens*; Sac. cerevisiae, *Saccharomyces cerevisiae*; Sal. enterica, *Salmonella enterica*; Mmv. hollandica, *Methanomethylovorans hollandica*; Mth. harundinacea, *Methanothrix harundinacea*; Mth. soehngenii, *Methanothrix soehngenii*.

**Figure S4**. Global transcriptional changes between the ∆*mreA* strain relative to WWM60 (wildtype or WT) during growth on acetate. In the volcano plot, genes with higher expression in the ∆*mreA* mutant have a positive log_2_(fold change) value. Genes with higher expression in the ∆*mreA* mutant have a negative log_2_(fold change) value. Values that meet the significant log_2_(fold change) in transcript abundance (q-value ≤ 0.01) are shaded black, while those that do not are shown as gray dots. Values associated with genes presented in Figure 2C and Figure 2D are shaded in other colors: *mreA* is shaded in white, acetate catabolism genes are shaded in red, and *fpo* genes are shaded in blue.

**Figure S5**. Promoter replacement for the *fpoA-O1* operon allows for tetracycline-inducible control of gene expression. (**A**) Graphical depiction of the promoter swap experiment used to replace the native *fpo* promoter with a tetracycline-inducible methyl-coenzyme M reductase promoter. Abbreviations: P*_fpo_*, *fpo* operon promoter; BRE, B-recognition element; TATA, TATA box; +1, transcription start site (gray arrow for native P*_fpo_* promoter, green arrow for P*_mcrB_*_(tetO4)_ promoter); T*_mcr_*, *mcr* operon terminator from *Methanosarcina acetivorans*; P*_mcrB_*_(tetO4)_*,* minimal *mcr* operon promoter from *Methanosarcina barkeri* with tetracycline binding site variant 4 from Guss, et al (2008); 5’ UTR, untranslated region at the 5’ of the transcript before *fpoA*. (**B**) Expression from relevant promoters and estimated expression from the P*_mcr_*_(tetO4)_ promoter which is used to overexpress *fpo’* genes in *M. acetivorans*. Transcript abundance of the first gene in the *mcr* and *fpo* operons (*mcrB* and *fpoA*, respectively) from the parent (WWM60 or wildtype, WT) are used as a proxy for promoter activity during growth on 50 mM TMA or 40 mM acetate (dark green bars). Transcript abundance is reported as fragments per kilobase per million reads (FPKM). Expression values on TMA are derived from the transcriptomic dataset reported by Downing, et al. (2023), and values on acetate are taken from the present study. Estimations for the expression level of the P*_mcr_*_(tetO4)_ promoter (hatched light green bars) were obtained by adjusting the P*_mcrB_* (Acetate) values by a factor of 0.27 based on: (i) the relative strength of the P*_mcrB_*_(tetO4)_ promoter compared to the P*_mcrB_*_(tetO1)_ promoter determined by Guss, et al. (2008), and (ii) the relative strength of the P*_mcrB_*_(tetO1)_ promoter compared to the P*_mcrB_* promoter determined by Chadwick, et al. (2024). The relative strength of induction at different concentrations of tetracycline is based on the dose response curves from Guss, et al. (2008) suggesting an ~75% strength for 25 µg/mL vs 100 µg/mL.

**Figure S6**. Promoter replacement of the *fpo* operon in the ∆*mmcA-rnf* background does not limit growth due to a lack of TetR. Growth curve ∆*mmcA-rnf* mutant (white triangles) and ∆*mmcA-rnf* P*_mcrB_*_(tetO4)_*-fpo* mutant strains supplemented with 25 µg/mL tetracycline (pink triangles) or 0 µg/mL tetracycline (purple triangles) in high-salt (HS) minimal media with 50 mM TMA. Four replicate tubes of each strain were used growth assays. Doubling times were not significantly different between strains (**Table S7**).
